# Memory retrieval in addiction: a role for miR-105-mediated regulation of D1 receptors in mPFC neurons projecting to the basolateral amygdala

**DOI:** 10.1186/s12915-017-0467-2

**Published:** 2017-12-27

**Authors:** Yanfang Zhao, Junfang Zhang, Hualan Yang, Dongyang Cui, Jiaojiao Song, Qianqian Ma, Wenjie Luan, Bin Lai, Lan Ma, Ming Chen, Ping Zheng

**Affiliations:** State Key Laboratory of Medical Neurobiology, Department of Neurology, Zhongshan Hospital, Collaborative Innovation Center for Brain Science, School of Basic Medical Sciences and Institutes of Brain Science, Fudan University, Shanghai, 200032 China

**Keywords:** Morphine, D1 receptors, Basolateral amygdala, Medial prefrontal cortex, miR-105, Opiate withdrawal memory

## Abstract

**Background:**

Drug addiction is a chronic brain disorder characterized by the compulsive use of drugs. The study of chronic morphine-induced adaptation in the brain and its functional significance is of importance to understand the mechanism of morphine addiction. Previous studies have found a number of chronic morphine-induced adaptive changes at molecular levels in the brain. A study from our lab showed that chronic morphine-induced increases in the expression of D1 receptors at presynaptic terminals coming from other structures to the basolateral amygdala (BLA) played an important role in environmental cue-induced retrieval of morphine withdrawal memory. However, the neurocircuitry where the increased D1 receptors are located and how chronic morphine increases D1 receptor expression in specific neurocircuits remain to be elucidated.

**Results:**

Our results show that chronic morphine induces a persistent increase in D1 receptor expression in glutamatergic terminals of projection neurons from the medial prefrontal cortex (mPFC) to the BLA, but has no influence on D1 receptor expression in projection neurons from the hippocampus or the thalamus to the BLA. This adaptation to chronic morphine is mediated by reduced expression of miR-105 in the mPFC, which results in enhanced D1 receptor expression in glutamatergic terminals of projection neurons from the mPFC to the BLA. Ex vivo optogenetic experiments show that a chronic morphine-induced increase in D1 receptor expression in glutamatergic terminals of projection neurons from the mPFC to the BLA results in sensitization of the effect of D1 receptor agonist on presynaptic glutamate release. mPFC to BLA projection neurons are activated by withdrawal-associated environmental cues in morphine-withdrawal rats, and overexpression of miR-105 in the mPFC leads to reduced D1 receptor induction in response to chronic morphine in glutamatergic terminals of the projection neurons from the mPFC to the BLA, and a reduction in place aversion conditioned by morphine withdrawal.

**Conclusions:**

These results suggest that chronic morphine use induces a persistent increase in D1 receptors in glutamatergic terminals of projection neurons from the mPFC to the BLA via downregulation of miR-105 in the mPFC, and that these adaptive changes contribute to environmental cue-induced retrieval of morphine withdrawal memory.

**Electronic supplementary material:**

The online version of this article (doi:10.1186/s12915-017-0467-2) contains supplementary material, which is available to authorized users.

## Background

Drug addiction is a chronic brain disorder characterized by the compulsive use of drugs [1]. The repeated use of addictive drugs, such as morphine, can result in increased drug craving, tolerance to morphine analgesia, and the expression of withdrawal syndromes when drug use is discontinued [2]. Moreover, chronic morphine-induced reward or withdrawal responses can be paired with neutral environmental stimuli. As a result of this pairing, the environmental neutral cues acquire a capability, on subsequent exposure, to elicit a retrieval of morphine reward or withdrawal memory in abstinent morphine addicts, leading to relapse [3]. Therefore, the study of chronic morphine-induced adaptive changes in the brain and their functional significance is of importance for understanding the mechanism of morphine addiction.

Previous studies have found a number of adaptive changes induced by chronic morphine at molecular levels in the brain such as changes in dopamine receptors [4], dopamine transporters [5], ∆FosB [6], brain-derived neurotropic factor [7], and opioid receptors [1]. Among them, a study from our lab showed that chronic morphine induced a significant increase in the expression of D1 receptors at presynaptic terminals coming from other structures to the basolateral amygdala (BLA), and this increase was shown to be relevant to environmental cue-induced retrieval of withdrawal memory because the intra-BLA injection of D1 receptor antagonist significantly inhibited conditioned place aversion (CPA) in morphine-withdrawal rats [8], a widely used animal model of the retrieval of opiate withdrawal memory [9, 10]. This result was consistent with other evidence for the importance of the BLA in environmental cue-induced retrieval of withdrawal memory. Schulteis et al. [11] reported that the BLA lesion using quinolinic acid significantly attenuated environmental cue-induced food aversion in morphine-withdrawal rats. However, the specific neurocircuitry involved was not established in these previous studies.

It is known that BLA receives glutamatergic projections from brain regions such as the medial prefrontal cortex (mPFC) [12, 13], the hippocampus [14], and the thalamus [15, 16]. The functions of these different projections are different [16], yet all these regions are important for memory retrieval. For example, in rats, the thalamus has been shown to be involved in fear memory retrieval [17], the hippocampus in reward memory retrieval [18], and the mPFC in withdrawal memory retrieval [19]. In this paper, we used a triple immunofluorescence method to investigate the influence of chronic morphine on the expression of D1 receptors in glutamatergic terminals of projection neurons from the mPFC, the hippocampus, or the thalamus to the BLA. We also studied the mechanism of the effect of chronic morphine on the expression of D1 receptors in identified glutamatergic projection neurons and its functional significance using optogenetics combined with multiple approaches. We found that chronic morphine selectively induced a persistent increase in the expression of D1 receptors in glutamatergic terminals of projection neurons from the mPFC to the BLA, and that this occurred as a result of downregulation of miR-105, a microRNA [20] which we show here modulates the expression of D1 receptors post-transcriptionally. We also demonstrate a behavioral impact of overexpressing miR-105 from lentiviral constructs injected into the mPFC, which led to reduced chronic morphine-induced D1 receptor expression in glutamatergic terminals of the projection neurons from the mPFC to the BLA (compared to control animals without overexpression of miR-105), and resulted in reduced CPA in morphine-withdrawal rats.

## Results

### Chronic morphine increases D1 receptor expression in glutamatergic terminals of projection neurons from the mPFC to the BLA, but has no influence on those of projection neurons from the hippocampus or the thalamus to the BLA

**Fig. 1:**
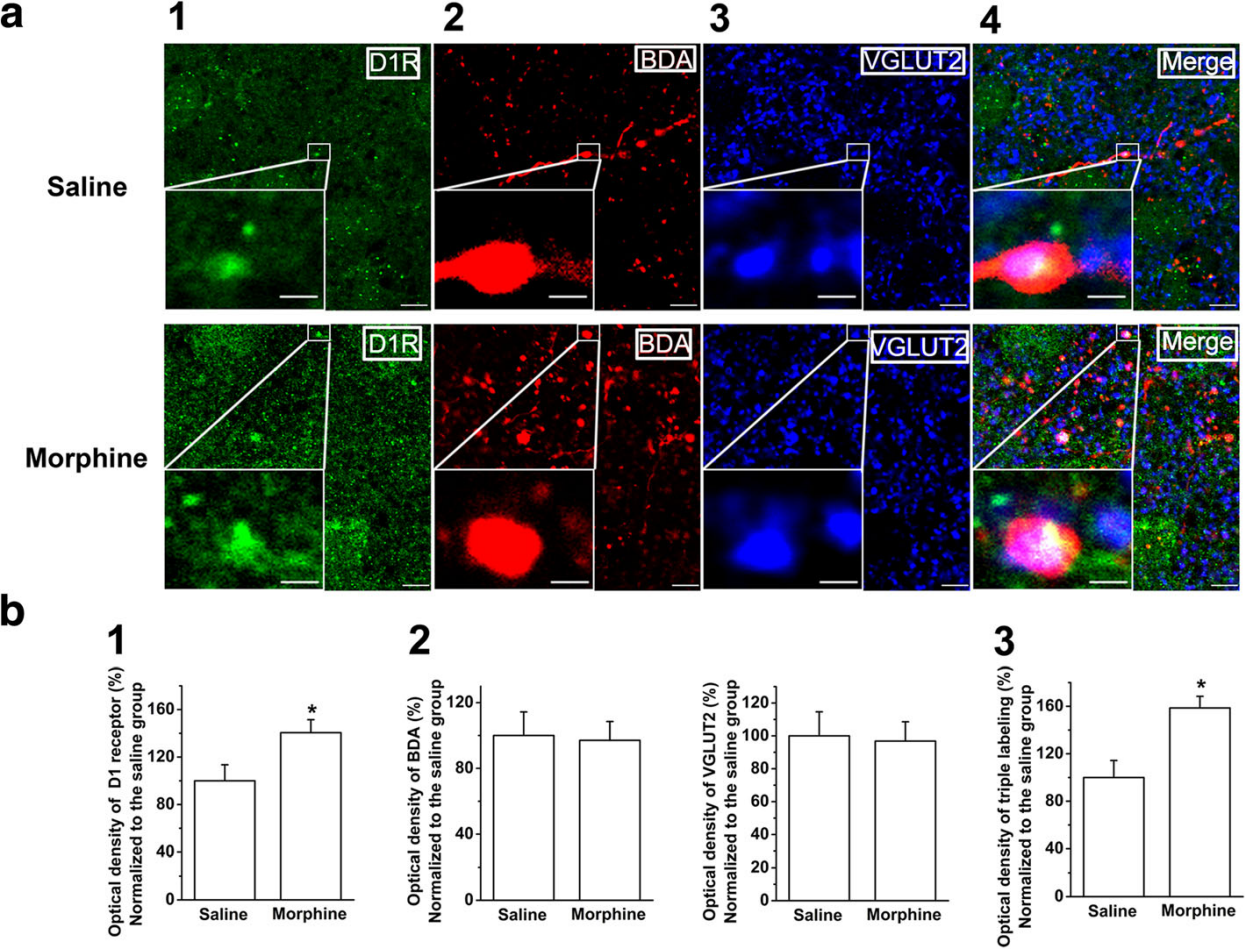
Effect of chronic morphine on D1 receptor expression in glutamatergic projection neurons from the mPFC to the BLA using triple-immunofluorescence method. **a** Top and bottom panels showed D1R staining (column 1, green color), BDA labeling (column 2, red color), glutamatergic terminal marker VGLUT2 immunoreactivity (column 3 blue color), and coexpression of D1R, BDA, and VGLUT2 (column 4, white color) in slices of the BLA in saline and chronic morphine group. The insets in figures are the magnified views marked with small white rectangles. Scale bar, 5 μm. In higher magnification of areas in the insets, the scale bar is 1 μm. **b** The averaged optical density of D1R (panel 1), BDA- and VGLUT2-labeled axon terminals (panel 2), and triple labeling clusters in the saline and morphine groups (panel 3) (n = 6, *P < 0.05, compared to saline group). Data are shown as the mean ± SEM

To study the effect of chronic morphine treatment on the expression of dopamine D1 receptor (D1R) in glutamatergic terminals of projection neurons from the mPFC to the BLA, we observed the influence of chronic morphine on the coexpression of D1R, vesicular glutamate transporters 2 (VGLUT2), and biotinylated dextran amine (BDA) in slices of the BLA. Here, we used BDA as an anterograde tracer because it was preferentially transported anterogradely and could label axons and terminals [21]. The top and bottom panels of Fig. 1a show D1R staining (column 1, green color, Fig. 1a), BDA labeling (column 2, red color, Fig. 1a), glutamatergic terminal marker VGLUT2 immunoreactivity (column 3, blue color, Fig. 1a), and coexpression of D1R, BDA, and VGLUT2 (column 4, white color, Fig. 1a) in slices of the BLA in the saline and chronic morphine groups. Figure 1b showed the fluorescence intensity of green, red, blue, and white color, which was quantified using MacBiophotonics Image J software in a series of stained sections from chronic morphine- or saline-treated animals. The results showed that chronic morphine did not change the fluorescence density of BDA (panel 2, Fig. 1b) and VGLUT2 (panel 2, Fig. 1b), but significantly increased the fluorescence density of D1 receptors (panel 1, Fig. 1b, saline group: 100.0 ± 13.5%, n = 6; morphine group: 140.5 ± 10.9%, n = 6, independent *t* test, *P* = 0.009, compared to saline group) and the coexpression of D1R, BDA, and VGLUT2 in the BLA (panel 3, Fig. 1b, saline group: 100.0 ± 14.3%, n = 6; morphine group: 158.6 ± 9.8%, n = 6, independent *t* test, *P* = 0.007, compared to saline group). This result suggested that chronic morphine could significantly increase the expression of D1 receptors in the BLA and in glutamatergic terminals of projection neurons from the mPFC to the BLA.

**Fig. 2:**
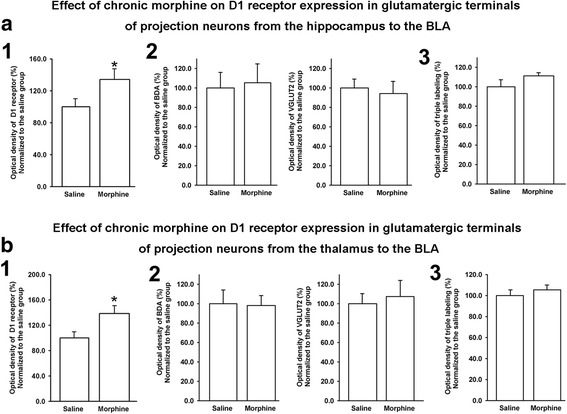
Effect of chronic morphine on D1 receptor expression in glutamatergic projection neurons from the hippocampus and the thalamus to the BLA using triple-immunofluorescence method. **a** Panel 1: the averaged optical density of D1R; panel 2: BDA- and VGLUT2-labeled axon terminals; panel 3: triple labeling clusters in saline and morphine groups (n = 9, *P < 0.05, compared to saline group). **b** The averaged optical density of D1R (panel 1), BDA-labeled axon terminals (panel 2), VGLUT2 (panel 3), and triple labeling clusters in saline and morphine groups (3) (n = 6, *P < 0.05, compared to saline group). Data are shown as the mean ± SEM

We also examined the influence of chronic morphine on the expression of D1 receptors in glutamatergic terminals of projection neurons from the hippocampus or the thalamus to the BLA using a similar method. Figure 2a shows the fluorescence intensity of green, red, blue, and white color quantified using MacBiophotonics Image J software in stained glutamatergic terminals of projection neurons from the hippocampus to the BLA sections from the saline and chronic morphine groups. The results indicate that chronic morphine did not change the fluorescence density of BDA (panel 2, Fig. 2a), VGLUT2 (panel 2, Fig. 2a), and the coexpression of D1R, BDA, and VGLUT2 in the BLA (panel 3, Fig. 2a, saline group: 100.0 ± 7.3%, n = 9; morphine group: 111.3 ± 3.3%, *n* = 9, independent *t* test, *P* = 0.175, compared to saline group), but significantly increased the fluorescence density of D1 receptors (panel 1, Fig. 2a, saline group: 100.0 ± 10.3%, *n* = 9; morphine group: 134.4 ± 13.3%, *n* = 9, independent *t* test, *P* = 0.013, compared to saline group). These results suggest that chronic morphine has no significant influence on the expression of D1 receptors in glutamatergic terminals of projection neurons from the hippocampus to the BLA, although it has an influence on the whole expression of D1 receptors in the BLA. In addition, Fig. 2b shows the fluorescence intensity of green, red, blue, and white color, which was quantified using MacBiophotonics Image J software in stained glutamatergic terminals of projection neurons from the thalamus to the BLA sections from the saline and chronic morphine groups. The results indicate that chronic morphine did not change the fluorescence density of BDA (panel 2, Fig. 2b), VGLUT2 (panel 2, Fig. 2b), and the coexpression of D1R, BDA, and VGLUT2 in the BLA (panel 3, Fig. 2b, saline group: 100 ± 5.5%, *n* = 6; morphine group: 105.5 ± 4.6%, *n* = 6, independent *t* test, *P* = 0.157, compared to saline group), but significantly increased the fluorescence density of D1 receptors (panel 1, Fig. 2b, saline group: 100.0 ± 9.8%, n = 6; morphine group: 139.3 ± 12.4%, *n* = 6, independent *t* test, *P* = 0.018, compared to saline group). These results suggest that chronic morphine had no significant influence on the expression of D1 receptors in glutamatergic terminals of projection neurons from the thalamus to the BLA, although it had an influence on the whole expression of D1 receptors in the BLA.

**Fig. 3:**
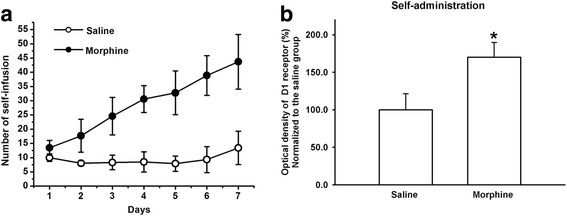
Effect of self-administration of morphine on D1 receptor expression in glutamatergic projection neurons from the mPFC to the BLA using triple-immunofluorescence method. **a** The self-infusion number during the period of morphine self-administration. **b** The averaged optical density of D1R in the glutamatergic terminals from the mPFC to the BLA in saline and morphine self-administration groups (n = 6, *P < 0.05, compared to saline group). Data are shown as the mean ± SEM

There are two kinds of drug administration paradigm in an animal addiction study, wherein one is a non-contingent (e.g., experimenter-administered) paradigm and the other is a contingent (self-administered drug) paradigm. Since human addiction involves drug self-administration, the validity of the latter model exceeds that of the former. However, the non-contingent model is still used because of the relative ease of drug delivery and the overlap in some of the biological changes elicited by contingent and non-contingent drug administration [22]. To examine whether contingent and non-contingent morphine exposure have a similar effect on D1 receptor expression in glutamatergic terminals of projection neurons from the mPFC to the BLA, we observed the effect of self-administration of morphine on D1 receptor expression in glutamate terminals of projection neurons from the mPFC to the BLA using the self-administration paradigm. The self-infusion number during the period of morphine self-administration is presented in Fig. 3a. Two-way ANOVA for repeated measures revealed that the self-infusion number had a treatment × time interaction effect (two-way ANOVA, *F*_(6, 60)_ = 47.66, *P* < 0.001). There was a significant difference between the number of self-infusions in the saline and morphine groups on day 7 (Bonferroni post hoc analysis, *t* = 12.75, *P* < 0.001). At 4 h after administration of the last test, D1R staining, BDA labeling, glutamatergic terminal marker VGLUT2 immunoreactivity, and coexpression of D1R, BDA and VGLUT2 were examined. The results showed that, similar to the experimenter-administered paradigm, self-administration of morphine also significantly increased the expression of D1 receptors in the BLA (saline self-administration group: 100 ± 13.8%, *n* = 6; morphine self-administration group: 145.8 ± 15.7%, *n* = 6, independent *t* test, *P* = 0.009, compared to saline self-administration group) and in glutamatergic terminals of projection neurons from the mPFC to the BLA (Fig. 3b, saline self-administration group: 100 ± 21.3%, *n* = 6; morphine self-administration group: 170.0 ± 19.6%, *n* = 6, independent *t* test, *P* = 0.002, compared to saline self-administration group).

To determine the potential relevance of chronic morphine-induced increases in the expression of D1 receptors in glutamatergic terminals of projection neurons from the mPFC to the BLA to the retrieval of opiate withdrawal memory, we examined whether the expression of D1 receptors in these glutamatergic terminals remained at a high level before measuring CPA in morphine-withdrawal rats. The results showed that D1 receptor expression in glutamatergic terminals of projection neurons from the mPFC to the BLA remained at a high level prior to CPA measurement in morphine-withdrawal rats (saline group before measuring CPA: 100 ± 16.7%, n = 6; chronic morphine group before measuring CPA: 147.2 ± 28.9%, n = 6, independent *t* test, *P* = 0.006, compared to saline group). This result suggests that the high expression of D1 receptors in glutamatergic terminals of projection neurons from the mPFC to the BLA may play a role in CPA.

### Chronic morphine inhibits the expression of miR-105 in the mPFC, which results in enhanced D1 receptor expression in glutamatergic terminals of projection neurons from the mPFC to the BLA

To study how chronic morphine treatment induced an increase in D1 receptor expression in glutamatergic terminals of projection neurons from the mPFC to the BLA, we firstly examined the effect of chronic morphine treatment on the expression of D1 receptor mRNA levels in projection neurons from the mPFC to the BLA using a retrograde tracing technique combined with a single-cell RT-PCR method. Fluorescent microspheres were injected into the BLA to label neurons from the mPFC to the BLA retrogradely. The cytoplasmic content of the labeled cell was harvested by a patch pipette to detect D1 receptor mRNA at a single cell level using a single-cell RT-PCR kit. The results showed that chronic morphine had no significant influence on D1 receptor mRNA level in mPFC-BLA projection neurons. The average percentage of the expression of D1 receptor mRNA in saline and chronic morphine groups was 100.0 ± 15.9% (*n* = 6) and 92.3 ± 11.4% (*n* = 6), respectively (independent *t* test, *P* = 0.697, compared to saline group).

**Fig. 4:**
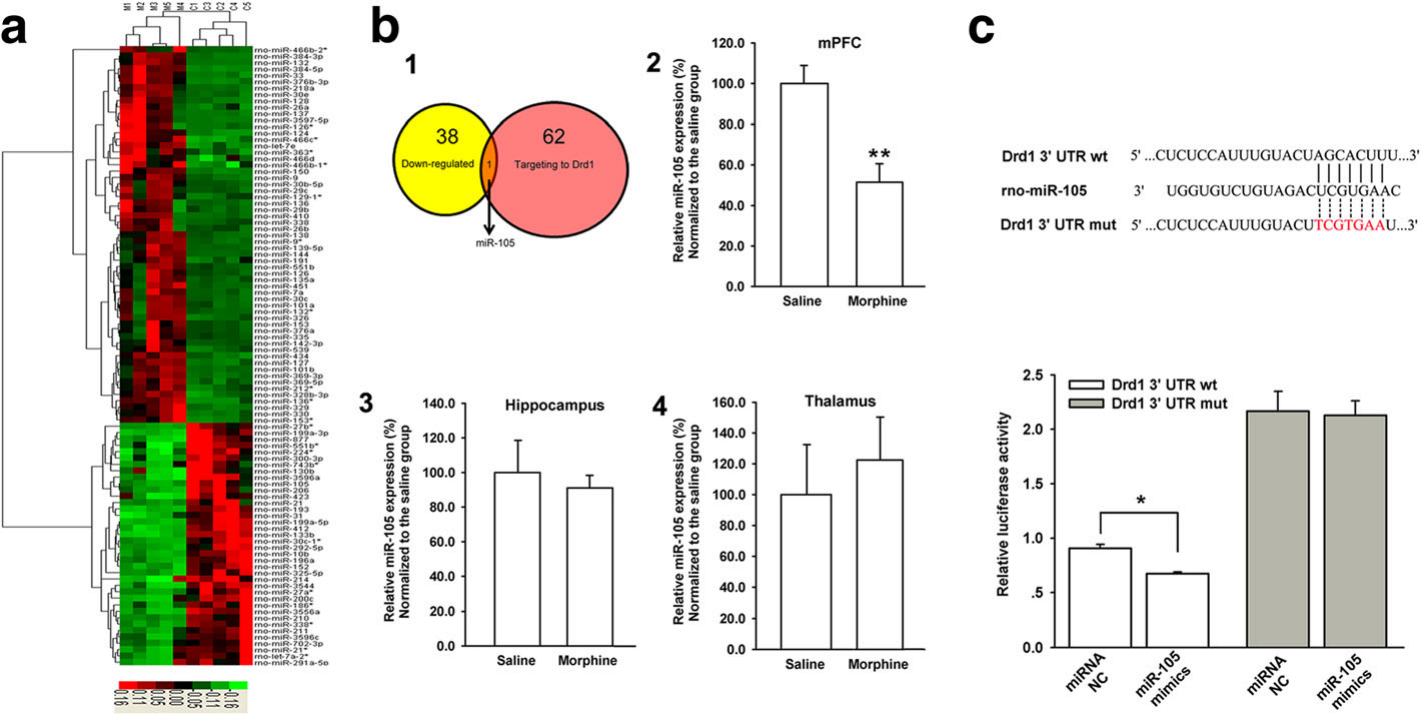
Effect of chronic morphine on the expression of microRNAs and the binding of miR-105 and D1 mRNA. **a** Expression of microRNAs in the mPFC in saline and chronic morphine groups using hierarchical clustering and heatmap. Green color on the heatmap indicates a decrease in microRNA expression level and red color indicates an increase in miRNA expression level as compared to saline group. **b** Panel 1: the overlap of 38 downregulated microRNAs and 62 microRNAs related to D1R gene (Drd1a) using bioinformatics analysis method in the chronic morphine group; panel 2: the averaged miR-105 expression in saline and chronic morphine groups by RT-PCR in the mPFC (n = 8, **P < 0.01, compared to saline group); panel 3: the averaged miR-105 expression in saline and chronic morphine groups by RT-PCR in the hippocampus (n = 4, *P* > 0.05, compared to saline group); panel 4: the averaged miR-105 expression in saline and chronic morphine groups by RT-PCR in the thalamus (n = 5, *P* > 0.05, compared to saline group). **c** Upper panel: bioinformatic analysis of the predicted miR-105 binding site in the 3′UTR of the Drd1 gene. Bottom panel: the luciferase activity with D1 mRNA 3′UTR wild-type (wt) in the presence of miR-105 miRNA NC and miR-105 mimics (n = 3, *P < 0.05, compared to miRNA NC group). Data are shown as the mean ± SEM

To explore whether chronic morphine regulated the expression of D1 receptors at post-transcriptional level, we examined the influence of chronic morphine on microRNA (miRNA) expression profile in the mPFC using miRNA microarray technology (Additional file 1). Figure 4a shows the hierarchical clustering analysis of differentially expressed miRNAs in the mPFC from morphine- and saline-treated rats. In this figure, the green color indicates a decrease in miRNA expression, whereas the red color indicates an increase in miRNA expression as compared to control. We found that 97 miRNAs (59 upregulated and 38 downregulated) were altered more than two-fold in the mPFC from chronic morphine-treated rats. Further bioinformatics analysis using the online prediction tools (Targetscan, http://www.targetscan.org and miRanda, http://www.microRNA.org) showed that miR-105 was the only one to be downregulated and related to the D1 receptor gene (*Drd1a*) (panel 1, Fig. 4b). The basic principle of predicting the relationship between miR-105 and the D1 receptor using these two tools follows the seed sequence of microRNA (2–7 nt) binding to the 3′-untranslated region (3′UTR) of the target gene through complementary base pairing. Therefore, we further investigated the role of miR-105 in chronic morphine-induced increases in D1 receptor expression in glutamatergic terminals of projection neurons from the mPFC to the BLA.

First, we used real-time RT-PCR to confirm the chronic morphine-induced decrease in expression of miR-105 in the mPFC. The result showed that chronic morphine indeed induced a decrease in the expression of miR-105 in the mPFC (panel 2, Fig. 4b, *n* = 8, paired *t* test, *P* = 0.002, compared to saline group). Interestingly, chronic morphine had no significant influence on the expression of miR-105 in the hippocampus and the thalamus. The average percentage of miR-105 level in the chronic morphine group was 91.0 ± 7.3%, showing no significant difference from that of the saline group (100.0 ± 18.5%) (panel 3, Fig. 4b, n = 4, paired *t* test, *P* = 0.743, compared to saline group) in the hippocampus. The average percentage of miR-105 level in chronic morphine group was 122.6 ± 27.7%, showing no significant difference with that of the saline group (100.0 ± 32.5%) (panel 4, Fig. 4b, *n* = 5, paired *t* test, *P* = 0.098, compared to saline group) in the thalamus. Then, we examined whether Drd1 was indeed a target of miR-105. Using Targetscan (http://www.targetscan.org) and miRanda (http://www.microRNA.org) prediction tools, it was predicted that miR-105 might bind to the 578–584 position of the 3′UTR of D1 mRNA via its 2–7 nucleotide seed sequence (upper panel of Fig. 4c). To confirm this binding, we constructed wt and mutant type (mut) 3′UTR plasmids of D1 mRNA and detected whether miR-105 could directly target D1 mRNA using a dual luciferase reporter assay method. The result showed that the miR-105 mimics reduced the luciferase activity of wt 3′UTR of D1 mRNA by 25.6 ± 2.0% (n = 3, paired *t* test, *P* = 0.009) in comparison with that of miRNA negative control (NC), but did not alter the luciferase activity of mut 3′UTR of D1 mRNA (n = 3, paired *t* test, *P* = 0.75, bottom panel of Fig. 4c). This result suggests that miR-105 can directly target 3′UTR of D1 mRNA.

**Fig. 5:**
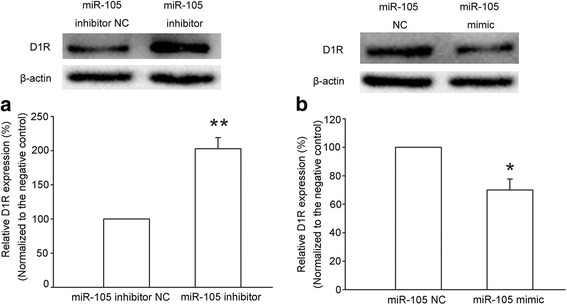
Effect of miR-105 inhibitor and mimic on the expression of D1 receptor (D1R) in mPFC-cultured neurons. **a** Levels of D1R in miR-105 inhibitor negative control (miR-105 inhibitor NC) and miR-105 inhibitor-transfected cultured mPFC neurons. Top panel: Immunoblots of D1R protein levels in miR-105 inhibitor NC and miR-105 inhibitor (40 μM)-transfected cultured mPFC neurons. Bottom panel: The average density of D1R bands after the normalization by β-actin protein in miR-105 inhibitor NC and miR-105 inhibitor-transfected-cultured mPFC neurons (n = 5, **P < 0.01, compared to miR-105 inhibitor NC group). **b** Levels of D1R in miR-105 negative control (miR-105 NC) and miR-105 mimic-transfected-cultured mPFC neurons. Top panel: Immunoblots of D1R protein levels in miR-105 NC and miR-105 mimic (20 μM)-transfected-cultured mPFC neurons. Bottom panel: The average density of the D1R bands after the normalization by β-actin protein in miR-105 NC and miR-105 mimic-transfected-cultured mPFC neurons (n = 5, *P < 0.05, compared to miR-105 NC group). Data are shown as the mean ± SEM

We further examined the influence of decreased miR-105 on D1 receptor expression. To do this, the miR-105 inhibitor (sequence complementary to miR-105) was transfected into primary cultured neurons of the mPFC. The result showed that miR-105 inhibitor-transfected cells exhibited an increase in D1 receptor expression (Fig. 5a, *n* = 5, paired *t* test, *P* = 0.0019, compared to miR-105 inhibitor NC group). In contrast, when we transfected the miR-105 mimic (same sequence as mature miR-105) to primary cultured neurons of mPFC, the expression of D1 receptors was suppressed (Fig. 5b, *n* = 5, paired *t* test, *P* = 0.03, compared to miR-105 NC group).

**Fig. 6:**
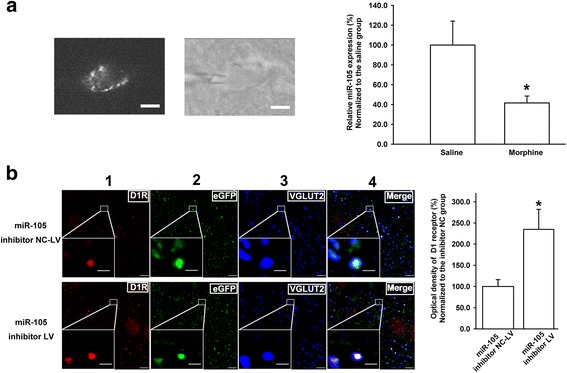
Effect of chronic morphine on miR-105 and influence of miR-105 inhibitor on D1 receptor expression in glutamatergic projection neurons from the mPFC to the BLA. **a** Left panel: neurons from the mPFC to the BLA labeled with fluorescent microspheres. Middle panel: harvesting the cytoplasmic contents of the labeled neuron via a patch pipette under visual control. Scale bar, 5 μm. Right panel: The averaged miR-105 expression in microsphere-labeled neurons in the mPFC in saline and morphine groups by single-cell RT-PCR (n = 5, *P < 0.05, compared to saline group). **b** Left panel: D1R staining (column 1, red color), eGFP labeling (column 2, green color), glutamatergic terminal marker VGLUT2 immunoreactivity (column 3 blue color), and coexpression of D1R, BDA, and VGLUT2 (column 4, white color) in slices of the BLA in miR-105 inhibitor NC-LV and miR-105 inhibitor-LV group. Right panel: The average optical density of D1R in glutamatergic terminals of projection neurons from the mPFC to the BLA in miR-105 inhibitor NC-LV and miR-105 inhibitor-LV groups (n = 5, *P < 0.05, compared to miR-105 inhibitor NC-LV group). The figure insets are the magnified views marked with small white rectangles; scale bar, 5 μm. In the insets of higher magnified areas the scale bar is 1 μm. Data are shown as the mean ± SEM

To study the role of chronic morphine-induced decrease of miR-105 in the expression of D1 receptors in the glutamatergic terminals of projection neurons from the mPFC to the BLA, we examined the effect of chronic morphine on the expression of miR-105 in projection neurons from the mPFC to the BLA using a retrograde tracing technique combined with single-cell RT-PCR. Fluorescent microspheres were injected into the BLA to label neurons from the mPFC to the BLA retrogradely. The left panel of Fig. 6a shows a labeled neuron in the mPFC. The cytoplasmic content of the labeled cell was harvested by a patch pipette (middle panel of Fig. 6a) to detect miR-105 at a single cell level using single-cell RT-PCR kit. The results showed that the average percentage of miR-105 level in the chronic morphine group was 41.6 ± 7.0%, which was significantly lower than that in the saline group (100.0 ± 24.1%) (right panel, Fig. 6a, *n* = 5, paired *t* test, *P* = 0.049, compared to saline group). This result suggests that chronic morphine induces a decrease in the expression of miR-105 in projection neurons from the mPFC to the BLA. On this basis, we observed the influence of the intra-mPFC injection of miR-105 inhibitor on D1 receptor expression in glutamatergic terminals of projection neurons from the mPFC to the BLA using a triple immunofluorescence staining method. The result showed that the intra-mPFC injection of miR-105 inhibitor could significantly increase the expression of D1 receptors in the glutamatergic terminals of projection neurons from the mPFC to the BLA (column 4, left panel, Fig. 6b, white color). The average percentage of the normalized D1 receptor intensity in the glutamatergic terminals of projection neurons from the mPFC to the BLA was 100.0 ± 16.2% in miR-105 inhibitor NC-lentiviral vector (LV) group (n = 5) and 234.8 ± 47.5% in miR-105 inhibitor-LV group (n = 5, independent *t* test, *P* = 0.028, compared to miR-105 inhibitor NC-LV group, right panel, Fig. 6b).

### Chronic morphine-induced increase in D1 receptor expression in glutamatergic terminals of projection neurons from the mPFC to the BLA results in sensitization to the effect of D1 receptor agonist on glutamate release

**Fig. 7:**
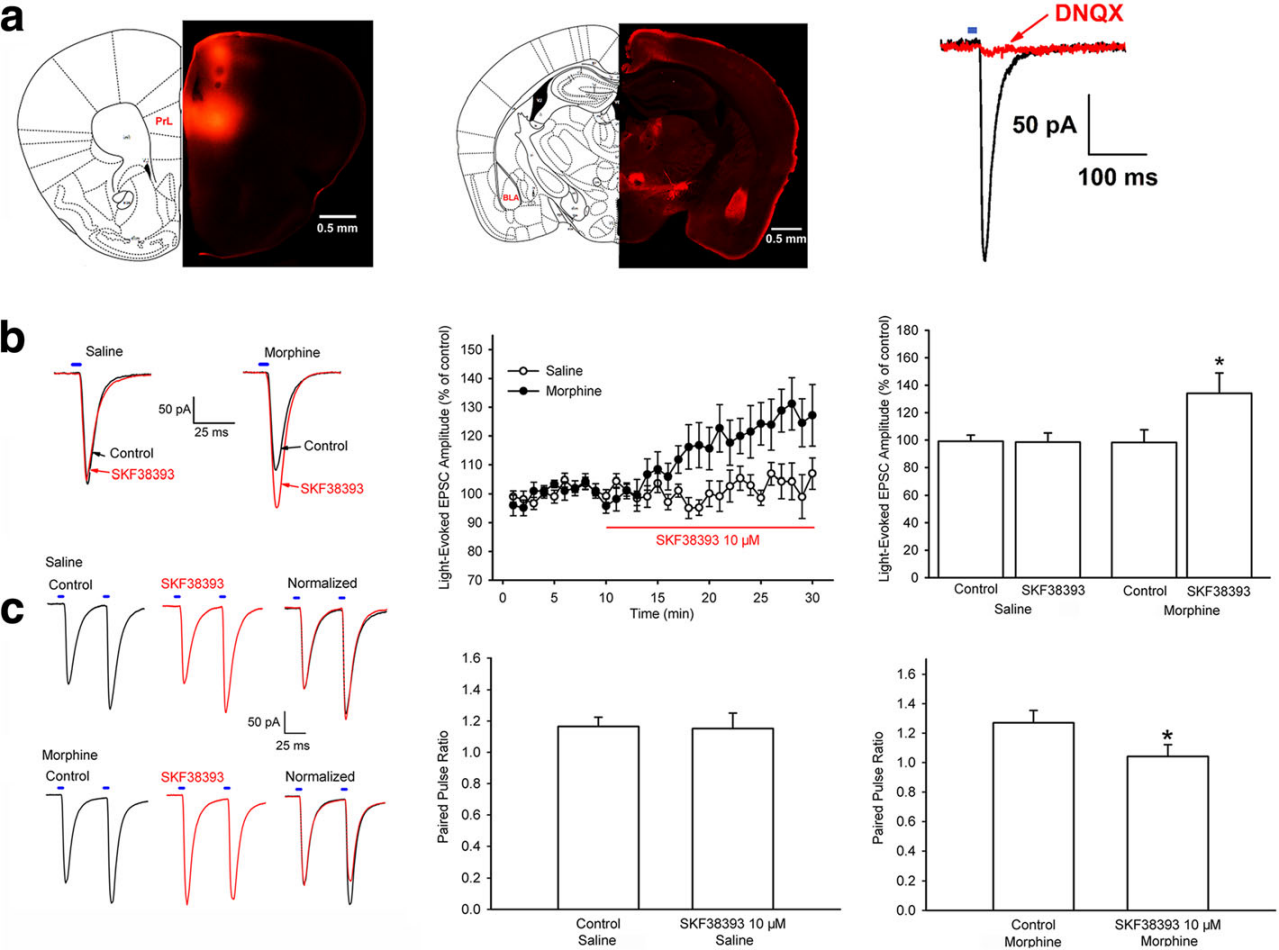
Effect of D1 receptor agonist SKF38393 on mPFC-to-BLA glutamatergic synaptic transmission under chronic morphine or saline conditions. **a** Left panel: Images of coronal brain slice showing expression of ChR2–mCherry (red color) 6 weeks after virus injection into the mPFC. Scale bar, 500 μm. Middle panel: Images of coronal brain slice showing strong ChR2–mCherry-positive fibers from the mPFC in the BLA 6 weeks after virus injection into the mPFC. Scale bar, 500 μm. Right panel: Light-evoked EPSCs recorded from BLA neurons after optical stimulation of mPFC-to-BLA fibers before and after bath application of DNQX. **b** Left panel: Representative traces recorded from BLA neurons after optical stimulation of mPFC-to-BLA fibers before and after bath application of 10 μM SKF38393 in response to 5 ms light pulses. Middle panel: Time course of light-evoked EPSC from BLA neurons before and after 10 μM SKF38393 in saline (n = 8 from 5 mice) and chronic morphine (n = 8 from 5 mice) group. Right panel: The averaged percentage of the effect of 10 μM SKF38393 on light-evoked EPSC in saline (n = 8 from 5 mice) and chronic morphine (n = 8 from 5 mice) groups. *P < 0.05, compared to before SKF38393. **c** Left panel: Representative traces recording of PPF from BLA neurons after a paired-pulse optical stimulation protocol before and after bath application of SKF38393 in saline and chronic morphine groups. Middle panel: Averaged PPF before and after 10 μM SKF38393 in saline group (n = 5 from 3 mice). Right panel: Averaged PPF before and after 10 μM SKF38393 in chronic morphine group (n = 5 from 3 mice). *P < 0.05, compared to before SKF38393. Data are shown as the mean ± SEM

To study the functional consequence of chronic morphine-induced increases in D1 receptor expression in glutamatergic terminals of projection neurons from the mPFC to the BLA, we used an optogenetic method to selectively stimulate excitatory fibers projecting from the mPFC to the BLA and then examined the effect of D1 receptor agonist SKF38393 on mPFC-to-BLA glutamatergic synaptic transmission under chronic morphine or saline conditions. The AAV-CaMKIIα-ChR2-mCherry virus was stereotaxically delivered into the mPFC; the left panel of Fig. 7a shows the expression of ChR2-mCherry in the mPFC after the injection of the virus. The virus was allowed to express for a minimum of 6 weeks in order to have sufficient opsin accumulation in the axons in the BLA (middle panel of Fig. 7a). Whole-cell recording was made in pyramidal cells of the BLA and optical stimulation of ChR2-mCherry-positive fibers from the mPFC to the BLA produced excitatory postsynaptic currents (EPSCs) in pyramidal cells of the BLA and these currents were blocked by bath application of AMPA receptor antagonist DNQX (10 μM) (right panel, Fig. 7a). This result demonstrated that these currents were AMPA receptor mediated. On this basis, we examined the effect of D1 receptor agonist SKF38393 on light-evoked EPSCs in the saline and chronic morphine groups. The left panel of Fig. 7b showed typical traces recorded from BLA neurons after the optical stimulation of the mPFC-to-BLA fibers before and at 15 min after SKF38393 (10 μM) addition in response to 5 ms light pulse in the saline and chronic morphine groups. From these raw traces, we could see that SKF38393 had no significant effect on the amplitude of light-evoked EPSCs in the saline group, but in the chronic morphine group, SKF38393 could significantly increase the amplitude of light-evoked EPSCs. The time course of EPSC response after SKF38393 in the two groups also showed a similar result (middle panel, Fig. 7b, n = 8, from five animals). The average amplitude of light-evoked EPSCs was 169.8 ± 8.7 pA before and 172.6 ± 11.6 pA at 15 min after SKF38393 addition in the saline group (right panel, Fig. 7b, n = 8, from five animals), but in the chronic morphine group, the average amplitude of light-evoked EPSCs increased from 180.6 ± 18.9 pA before to 234.9 ± 26.1 pA at 15 min after SKF38393 addition (right panel, Fig. 7b, n = 8, from five animals, one-way ANOVA, *F*_(3, 28)_ = 3.001, *P* = 0.047).

To investigate the effect of SKF38393 on presynaptic glutamate release, we observed the influence of SKF38393 on paired-pulse facilitation (PPF) of light-evoked EPSCs in the saline and chronic morphine groups. PPF, measured as the ratio of EPSC amplitude in response to two successive stimulation pulses, is a frequently used parameter to monitor presynaptic glutamate release [23]. The results showed that, in the saline group, SKF38393 had no effect on PPF, but in the chronic morphine group, after the addition of SKF38393, the first EPSC was increased by 31.5 ± 3.3% (*n* = 5, from three animals), whereas the second synaptic response was decreased by 1.6 ± 4.3% (*n* = 5, from three animals). Therefore, the superimposition of the two traces normalized to the first EPSC at 15 min after SKF38393 addition in the saline and chronic morphine groups revealed that PPF did not change after SKF38393 addition in the saline group, but decreased after SKF38393 addition in the chronic morphine group (left panel, Fig. 7c). The average PPF was 1.2 ± 0.1 before and 1.2 ± 0.1 at 15 min after SKF38393 addition in the saline group (middle panel, Fig. 7c, *n* = 5, from three animals, paired *t* test, *P* = 0.904, compared to control before SKF38393). However, the average PPF was decreased from 1.3 ± 0.1 to 1.0 ± 0.1 at 15 min after SKF38393 addition in the chronic morphine group (right panel, Fig. 7c; *n* = 5, from three animals, paired *t* test, *P* = 0.0299, compared to control before SKF38393). This result suggests that the D1 receptor agonist SKF38393 has no significant effect on glutamate release from the mPFC-to-BLA fibers in pyramidal cells of the BLA under normal conditions, but after chronic morphine, it can significantly increase it.

**Fig. 8:**
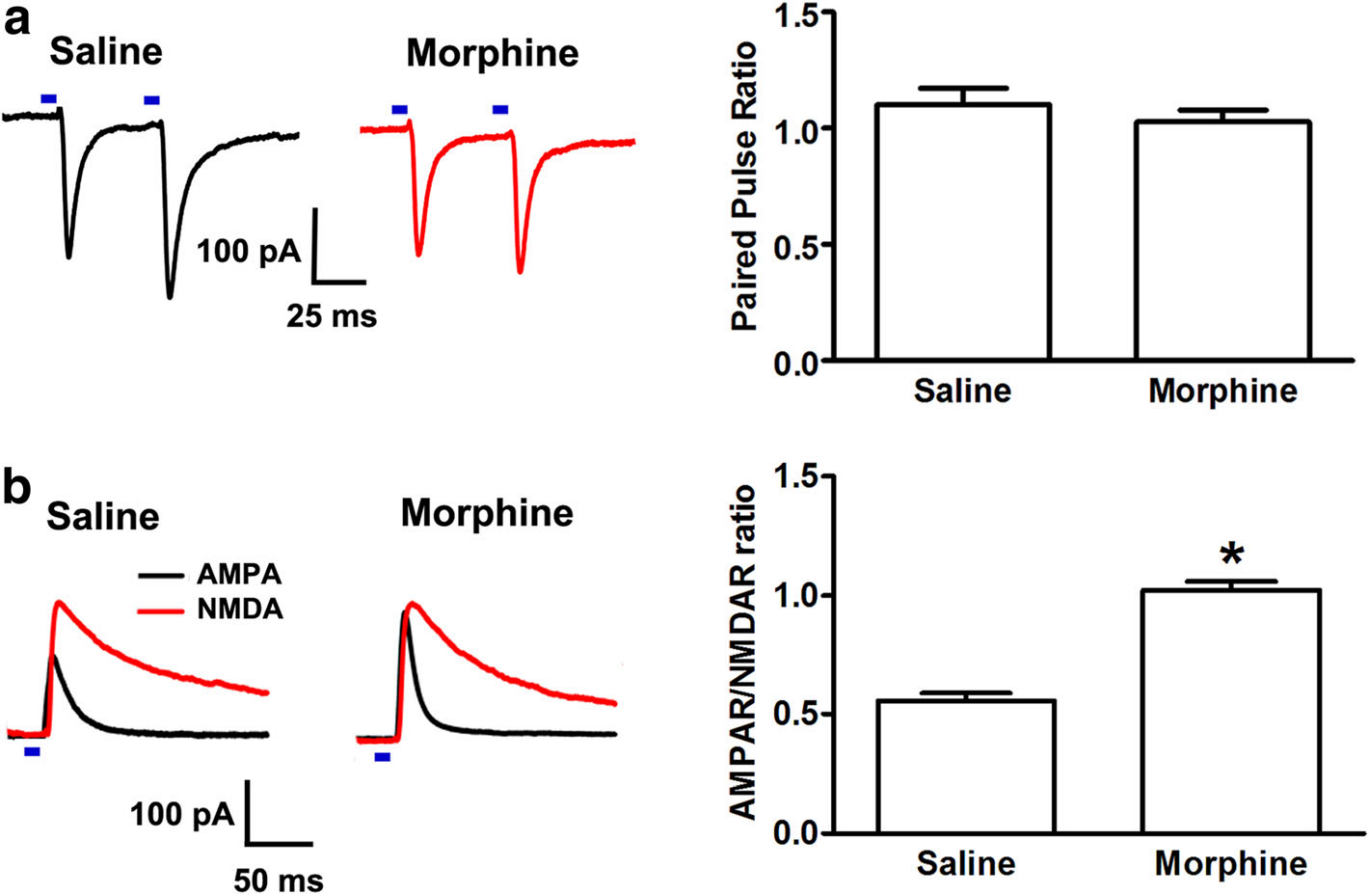
Influence of chronic morphine on basic parameters of excitatory synaptic transmission between mPFC-to-BLA fibers and pyramidal cells of the BLA. **a** Effect of chronic morphine on PPF of light-evoked EPSCs between mPFC-to-BLA fibers and pyramidal cells of the BLA. Left panel: Representative traces of EPSCs induced by a paired-pulse optical stimulation in saline and chronic morphine groups. Right panel: Average PPF of EPSCs in saline and chronic morphine groups (n = 8, independent *t* test, *P* > 0.05, compared with saline group). **b** Effect of chronic morphine on AMPAR/NMDAR ratio between mPFC-to-BLA fibers and pyramidal cells of the BLA. Left panel: Representative traces of light-evoked AMPAR and NMDAR currents recorded from pyramidal cells of the BLA after optical stimulation of mPFC-to-BLA fibers in saline and chronic morphine groups. Right panel: Average AMPAR/NMDAR ratio in BLA neurons from mPFC-to-BLA fibers in saline and chronic morphine groups (n = 8, independent *t* test, *P < 0.05, compared to saline group). Data are shown as the mean ± SEM

We also examined the influence of chronic morphine on the basic parameters of excitatory synaptic transmission between mPFC-to-BLA fibers and pyramidal cells of the BLA. We used PPF as the index of presynaptic glutamate release as described above and the ratio of light-evoked AMPAR-mediated EPSCs to NMDAR-mediated EPSCs (AMPAR/NMDAR ratio) as the index of glutamatergic synaptic strength [24]. The results showed that chronic morphine had no significant influence on the PPF of light-evoked EPSCs, but increased the AMPAR/NMDAR ratio. The average PPF in the chronic morphine group was 1.03 ± 0.05, with no significant difference with that of the saline group (1.10 ± 0.07) (right panel, Fig. 8a; n = 8, independent *t* test, *P* = 0.409, compared to saline group). The average AMPAR/NMDAR ratio in the chronic morphine group was 1.02 ± 0.04, which was significantly increased compared to the saline group (0.56 ± 0.03) (right panel, Fig. 8b; *n* = 8, independent *t* test, *P* < 0.001, compared to saline group). These results suggest that chronic morphine may not induce a direct change in glutamate release from the mPFC-to-BLA fibers in pyramidal cells of the BLA, but may cause a change in glutamatergic synaptic strength.

### Withdrawal-associated environmental cues can activate mPFC-to-BLA projection neurons in morphine-withdrawal rats

**Fig. 9:**
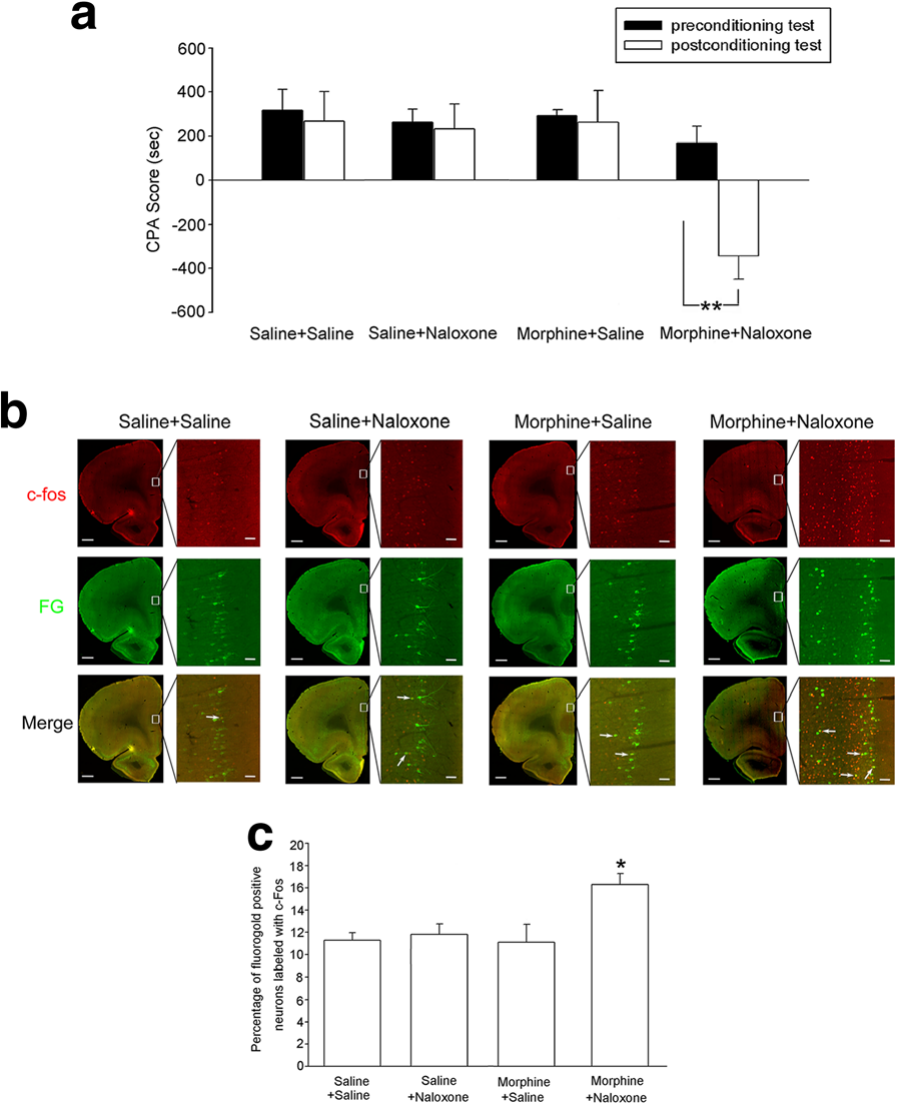
Effect of withdrawal-associated environmental cues on the activity of mPFC projection neurons to the BLA. **a** Averaged CPA scores in saline + saline, saline + naloxone, chronic morphine + saline, and chronic morphine + naloxone groups (*n* = 6, **P < 0.01, compared to preconditioning test in chronic morphine + naloxone group). **b** Effect of withdrawal-associated environmental cues on the expression of c-Fos in mPFC projection neurons to the BLA. Up line: c-fos positive cells (red color) in the mPFC in saline + saline, saline + naloxone, chronic morphine + saline, or chronic morphine + naloxone group. Middle line: FG-labeled neurons (green colored) in the mPFC in saline + saline, saline + naloxone, chronic morphine + saline, or chronic morphine + naloxone groups. Bottom line: co-labeling of c-Fos and FG (yellow color) in the mPFC in saline + saline, saline + naloxone, morphine + saline, or morphine + naloxone groups. Scale bar, 500 μm. mPFC regions enclosed by white boxes were shown in a higher magnification in right square images; scale bar, 50 μm. **c** The average percentage of the co-labeled positive neurons with FG and c-Fos in the mPFC in saline + saline, saline + naloxone, chronic morphine + saline, or chronic morphine + naloxone groups. *n* = 6, *P < 0.05, compared to saline + saline group. Data are shown as the mean ± SEM

To examine whether withdrawal-associated environmental cues could activate mPFC-to-BLA projection neurons, we used a precipitated withdrawal model. In this conventional model of withdrawal, opiate receptor antagonist naloxone is administered to morphine-dependent animals in order to precipitate withdrawal syndromes and for these to occur more reliably [25]. The procedure for the CPA test was similar to that described previously [26]. The rats were given a pre-test to assess their baseline place preference. Then, the rats were confined to withdrawal-paired compartments based on their baseline place preference in the training phase. The post-test was performed by re-exposure of rats to the chamber to freely explore all three compartments. Rats were divided four groups as saline + saline, saline + naloxone, chronic morphine + saline, and chronic morphine + naloxone. The results showed that the rats in the chronic morphine + naloxone group exhibited a strong aversion to withdrawal-paired compartment and thus spent less time in the withdrawal-paired compartment during the post-test than during the pre-test, producing an increase in ‘aversion score’ (CPA score), whereas rats in other groups did not exhibit a significant aversion to either compartment. The average CPA score in the chronic morphine + naloxone group was −342.1 ± 107.2 s (Fig. 9a, *n* = 6, two-way ANOVA, Bonferroni post hoc analysis, *F*_(3, 40)_ = 7.268, *P* = 0.0025), but in the saline + saline, saline + naloxone, and chronic morphine + saline groups, the rats had no significant aversive responses for the two compartments (Fig. 9a, *n* = 6). On this basis, we examined the effect of withdrawal-associated environmental cues on the expression of c-Fos in mPFC-to-BLA projection neurons in the saline + saline, saline + naloxone, chronic morphine + saline, and chronic morphine + naloxone groups using a retrograde tracing technique combined with c-Fos detecting method. Fluorogold (FG) was injected into the BLA to label neurons from the mPFC to the BLA retrogradely. The effect of withdrawal-associated environmental cues on the expression of c-Fos in FG-labeled neurons of the mPFC was examined. The upper line in Fig. 9b showed c-Fos expression (red color) and the middle line showed FG-labeled neurons (green color) of the mPFC in these four groups. The bottom line of Fig. 9b showed the co-labeling (yellow color) of c-Fos and FG. The co-labeling of c-Fos and FG significantly increased in the chronic morphine + naloxone group when rats were exposed to withdrawal-associated environmental cues. The average percentage of c-Fos positive neurons labeled with FG in the chronic morphine + naloxone group was 16.3 ± 1.0%, which was significantly higher than that in the saline + saline (11.3 ± 0.7%), saline + naloxone (11.8 ± 1.0%), or chronic morphine + saline (11.1 ± 1.6%) groups (*n* = 6, one-way ANOVA, Bonferroni post hoc analysis, *F*_(3, 20)_ = 4.482, *P* = 0.03, Fig. 9c). This result suggests that withdrawal-associated environmental cues can activate mPFC-to-BLA projection neurons in morphine-withdrawal rats.

### Overexpression of miR-105 in the mPFC leads to suppression of D1 receptor expression in glutamatergic terminals of the projection neurons from the mPFC to the BLA, and a reduction in CPA in morphine-withdrawal rats

**Fig. 10:**
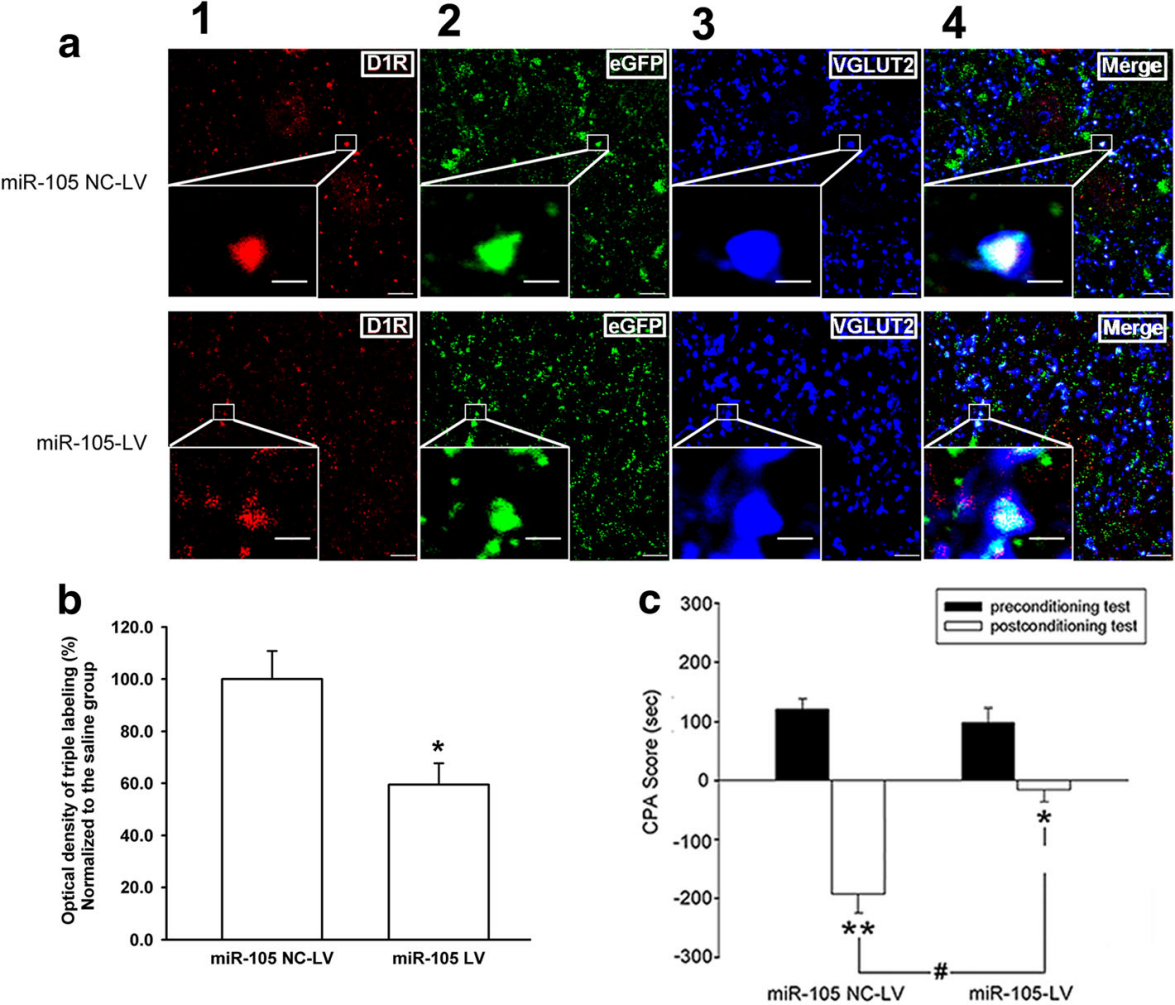
Influence of the overexpression of miR-105 on the expression of D1 receptors (D1R) in glutamatergic projection neurons from the mPFC to the BLA and the effect of the overexpression of miR-105 on CPA in morphine-withdrawal rats. **a** Top and bottom panels showed D1R staining (column 1, red color), eGFP labeling (column 2, green color), glutamatergic terminal marker VGLUT2 immunoreactivity (column 3, blue color), and coexpression of D1R, eGFP, and VGLUT2 (column 4, white color) in slices of the BLA in miR-105 NC-LV and miR-105-LV groups. The figure insets are the magnified views marked with small white rectangles. Scale bar, 5 μm. In higher magnification of areas in the insets the scale bar is 1 μm. **b** The average D1R expression intensity in glutamatergic projection neurons from the mPFC to the BLA in miR-105 NC-LV and miR-105-LV groups (n = 5, *P < 0.05, compared to miR-105 NC-LV group). **c** Effect of overexpression of miR-105 on CPA in morphine-withdrawal rats. *n* = 6, **P < 0.01, compared to preconditioning test in miR-105 NC-LV group. *P < 0.05, compared to preconditioning test in miR-105-LV group. ^#^ *P* < 0.05, compared to post-conditioning test in miR-105 NC-LV group. Data are shown as the mean ± SEM

To test the functional relevance of the morphine-induced suppression of miR-105 in the mPFC we opposed this by intra-mPFC injection of a LV containing a miR-105 precursor, injecting a control group of rats with the eGFP-labeled LV alone. This intervention was performed 3–4 weeks before morphine administration and we found that over-expressing miR-105 in the mPFC by these means significantly decreased the expression of D1 receptors in glutamatergic terminals of projection neurons from the mPFC to the BLA (relative to the control group), as assessed after morphine treatment using the triple immunofluorescence staining method. The top and bottom panels of Fig. 10a show D1R staining (column 1, Fig. 10a, red color), eGFP labeling (column 2, Fig. 10a, green color), glutamatergic terminal marker VGLUT2 immunoreactivity (column 3, Fig. 10a, blue color), and coexpression of D1R, eGFP, and VGLUT2 (column 4, Fig. 10a, white color) in slices of the BLA in the control group of rats injected with LV alone (miR-105 NC-LV) and the group injected with the miR-105 precursor (miR-105-LV). The average percentage of the normalized D1 receptor intensity in glutamatergic terminals of projection neurons from the mPFC to the BLA was 100.0 ± 10.8% in the miR-105 NC-LV group (*n* = 5) and 59.5 ± 8.3% in the miR-105-LV group (*n* = 5, independent *t* test, *P* = 0.018, compared to miR-105 NC-LV group, Fig. 10b). We then examined the influence of over-expressing miR-105 on environmental cue-induced place aversion in morphine-withdrawal rats. The results showed that, in the group of rats that had previously received the intra-mPFC injection of miR-105 overexpression lentivirus, the post-conditioning test CPA score was significantly lower (miR-105-LV group, −18.7 ± 23.9 s, *n* = 6) than that in the control group injected with the LV alone (miR-105 NC-LV group, −193.8 ± 35.1 s, *n* = 6) (two-way ANOVA, Bonferroni post hoc analysis, *F*_(1, 21)_ = 8.67, *P* = 0.007, compared to post-conditioning test CPA score in miR-105 NC-LV group, Fig. 10c). This result suggests that a chronic morphine-induced increase in D1 receptor expression in glutamatergic terminals of projection neurons from the mPFC to the BLA contributes to CPA in morphine-withdrawal rats. Nevertheless, miR-105 also has other targets that may affect these results. However, the present known targets of miR-105 are mainly those involved in tumor behavior and malignant progression [27–29] such as mRNAs of SOX9, SUZ, and NCOA1. Moreover, the results presented in Fig. 6 herein showed that a chronic morphine-induced increase in D1 receptor expression in glutamatergic terminals of projection neurons from the mPFC to the BLA resulted in a sensitization of the effect of D1 receptor agonist on glutamate release. Further, our pervious study showed that the intra-BLA injection of D1 receptor antagonist significantly inhibited CPA in morphine-withdrawal rats [8]. This evidence further supports the notion that D1 receptors in glutamatergic terminals of projection neurons from the mPFC to the BLA contribute to CPA in morphine-withdrawal rats, although we cannot completely eliminate the contributions of other targets of miR-105 in this behavior.

## Discussion

The main findings of this study are, firstly, that before the re-exposure to withdrawal-paired environmental cues, chronic morphine induces a persistent increase in D1 receptor expression in glutamatergic terminals of projection neurons from the mPFC to the BLA, but has no influence on those from the hippocampus and the thalamus to the BLA. Secondly, chronic morphine inhibits the expression of miR-105 in the mPFC, which results in an enhanced D1 receptor expression in glutamatergic terminals of projection neurons from the mPFC to the BLA. Thirdly, a chronic morphine-induced increase in D1 receptor expression in glutamatergic terminals of projection neurons from the mPFC to the BLA results in a sensitization of the effect of D1 receptor agonist on glutamate release. Fourthly, withdrawal-associated environmental cues activate mPFC-to-BLA projection neurons in morphine-withdrawal rats. Finally, overexpression of miR-105 in the mPFC leads to suppression of D1 receptor expression in glutamatergic terminals of the projection neurons from the mPFC to the BLA, and a concomitant reduction in CPA in morphine-withdrawal rats.

The mechanism underlying the effect of chronic morphine on the expression of D1 receptors in glutamatergic terminals of projection neurons from the mPFC to the BLA may occur at transcriptional or post-transcriptional level in the soma of the mPFC. At transcriptional level, our results showed that chronic morphine treatment had no significant influence on D1 receptor mRNA levels in mPFC neurons that projected to the BLA. However, at the post-transcriptional level, our results showed that chronic morphine inhibited the expression of miR-105 in the mPFC, resulting in enhanced D1 receptor expression in glutamatergic terminals of projection neurons from the mPFC to the BLA. miR-105 belongs to a region of the GABRA3A gene located on the X chromosome [20]. The precursor of miR-105 resides on intron I of the GABRA3A gene [30]. After being transcribed, cleaved, and exported from the nucleus to the cytoplasm, the pre-miRNA forms the mature miR-105, which then binds to specific sequences in the 3′UTRs of target mRNAs via its seed sequences (2–7 nucleotides) to degrade or inhibit the translation of mRNA. Previous studies reported that miR-105 might target several mRNAs, such as mRNAs of SOX9, SUZ, and NCOA1, which are involved in tumor behavior and malignant progression [27–29]. Here, we found that the D1 receptor was another target of miR-105. To our knowledge, this is the first report about the modulatory effect of miR-105 on the expression of D1 receptors. Interestingly, we found that chronic morphine had no significant influence on the expression of miR-105 in the hippocampus and the thalamus. These findings are consistent with the result that chronic morphine has no effect on D1 receptor expression in glutamatergic terminals in projection neurons from the hippocampus or the thalamus to BLA.

Previous studies have reported that, in opiate-dependent individuals, withdrawal-paired environmental cues could activate dopamine neurons in the ventral tegmental area (VTA) and promote dopamine release in the BLA [31, 32]. However, how the released dopamine acts in the BLA remains unknown. The present study shows that, before the re-exposure to withdrawal-paired environmental cues, chronic morphine induced an increase in D1 receptor expression in glutamatergic terminals of projection neurons from the mPFC to the BLA, which resulted in an enhanced glutamate release by D1 receptor agonists. Therefore, it is possible that, when opiate-withdrawal individuals are re-exposed to withdrawal-paired environmental cues, the cues promote glutamate release from glutamatergic terminals of projection neurons from the mPFC to the BLA by first activating dopamine neurons in the VTA, then promoting dopamine release in the BLA, and finally activating D1 receptors in the glutamatergic terminals of this projection neuron. This statement is consistent with the importance of D1 receptors in environmental cue-induced reinstatement of drugs. For example, Bossert et al. [33] reported that the systemic injection of D1 receptor antagonist SCH 23390 attenuated environmental cue-induced reinstatement of heroin seeking. Tingakee et al. [34] proposed that dopamine D1 receptors were not critical for opiate reward but can mediated opiate memory retrieval in a state-dependent manner. Additionally, these results are consistent with our previously published data indicating that chronic morphine-induced increases in D1 receptor expression in the BLA result in an enhanced presynaptic glutamate release, which subsequently switches the effect of dopamine on the excitatory synaptic transmission in pyramidal cells of BLA from inhibition to excitation, as well as that the intra-BLA injection of D1 receptor antagonist canceled environmental cue-induced place aversion in morphine-withdrawal rats [8].

Previous studies reported that, in opiate-withdrawal individuals, opiate-related cues could also activate the mPFC [35]. Moreover, Fuchs et al. [36] reported that mPFC inactivation could abolish environmental cue-induced reinstatement of cocaine-seeking behavior. However, the nature of the projection neurons in the mPFC are activated by withdrawal-paired environmental cues remains unknown. The present study showed that withdrawal-paired environmental cues could activate glutamatergic projection neurons from the mPFC to the BLA at the soma of these projection neurons. Moreover, in combination with the above discussion, it is possible that, when opiate-withdrawal individuals are re-exposed to opiate-related cues, the cues might produce a dual activation of the glutamatergic projection neurons from the mPFC to the BLA; on the one hand, the cues activate the soma of glutamatergic projection neurons in the mPFC and, on the other, they activate the terminals from the mPFC to the BLA by first activating dopamine neurons in VTA, then promoting dopamine release in BLA, and finally activating the D1 receptors in the glutamatergic projection neurons. The consequence of this dual activation on the glutamatergic projection neurons from the mPFC to the BLA may induce a retrieval of withdrawal memory in its downstream neurons. Interestingly, Pendyam et al. [37] proposed that projection neurons from the mPFC to the BLA also participate in the expression of fear memory, whereas Otis et al. [38] reported that projection neurons from the dorsomedial prefrontal neurons to the striatum are involved in conditioned reward-seeking behavior. In addition, Augur et al. [39] reported that chemogenetic activation of the ventromedial prefrontal cortex (mainly infralimbic cortex) and projection neurons of the ventromedial prefrontal cortex to the nucleus accumbens could reduce cue-induced reinstatement of cocaine seeking. Notably, prior to drug-related cue activation of the mPFC, drugs of abuse could already have caused abnormalities in the mPFC such as increases in excitatory presynaptic release, PPF, and increased AMPA receptor transmission [40].

Notably, when we checked the influence of withdrawal-paired environmental cues on the expression of c-Fos in glutamatergic projection neurons from the mPFC to the BLA, in addition to co-labeling neurons of c-Fos and FG, which represent projection neurons from the mPFC to the BLA, there were some c-fos positive neurons without FG co-labeling. This phenomenon suggests that withdrawal-paired environmental cues might activate mPFC output neurons that project to other regions, indicating the complexity of the involvement of the mPFC in environmental cue-induced retrieval of withdrawal memory. This situation also occurred when the mPFC participated in reward-related responses. For example, Otis et al. [38] reported that bidirectional optogenetic manipulation of corticostriatal neurons promoted conditioned reward-seeking behavior, while the activity in corticothalamic neurons suppressed both the acquisition and expression of conditioned reward seeking. Ferenczi et al. [41] proposed that the mPFC modulated the expression of reward-seeking behavior by regulating the dynamic interactions between specific distant subcortical regions.

The BLA has been demonstrated to play an important role in the retrieval of withdrawal memory. Re-exposure to withdrawal-paired environment cues induced an increase in the expression of c-Fos, a marker of neuronal activation, in the BLA [32]. The BLA lesion using quinolinic acid significantly attenuated the cue-induced place aversion in morphine-withdrawal rats [11]. Moreover, BLA output neurons showed an increase in the expression of plasticity-related *Arc* gene after re-exposure to the withdrawal-paired environment [42], suggesting that there might be a retrieval of withdrawal memory signals in BLA output neurons. Therefore, it is possible that the downstream neurons of the mPFC to BLA projection neurons, which exhibit a retrieval of withdrawal memory signals, are BLA output neurons that have an environmental cue-induced high expression of *Arc*.

## Conclusions

We conclude that chronic morphine-induced persistent increases in the expression of D1 receptors in glutamatergic terminals of projection neurons from the mPFC to the BLA via downregulation of miR-105 contributes to environmental cue-induced retrieval of morphine withdrawal memory. However, how chronic morphine induces the downregulation of miR-105 remains to be elucidated. The further study of its signaling pathways would provide new targets to interfere with chronic morphine-induced increased in the expression of D1 receptors in glutamatergic terminals of projection from the mPFC to the BLA.

## Methods

### Chronic morphine treatment

Male Sprague Dawley rats (220–250 g) and male C57BL/6 J mice (3 weeks old) were treated with morphine according to previously described procedures [43]. Briefly, morphine dependence was induced in rats and mice by repeated intraperitoneal injections of morphine twice daily at 08.00 AM and 19.00 PM. Morphine doses were progressively increased from 10 mg/kg to 40 mg/kg: first day 2 × 10 mg/kg, second day 2 × 20 mg/kg, third day 2 × 30 mg/kg, fourth and fifth days 2 × 40 mg/kg. Control rats and mice were treated with saline following the same procedure.

### Anterograde tracing

After the rats were anesthetized with chloral hydrate (400 mg/kg, i.p.) and secured in a stereotaxic device (Stoelting), anterograde tracing marker biotinylated dextran amine (BDA, 10,000 MW, SP-1130, Vector Lab, 10% dissolved in 0.1 M PH 7.4 phosphate buffer) was injected into the mPFC (AP, +3.0 mm; ML, ±0.5 mm; DV, −3.5 mm), the hippocampus (AP, −5.2 mm; ML, ±5 mm; DV, −8.5 mm), and the thalamus (AP, −5.2 mm; ML, ±2.9 mm; DV, −5.7 mm) based on the atlas of Paxinos and Watson (2005) [44] in a 0.5-μL volume in each side of the brain and retained in place for an additional 10 min to optimize diffusion. Following the BDA tracing in axons for 7–10 days, rats were treated with morphine as described above, perfused with saline, and followed by 4% paraformaldehyde in PB. After 4 h, they were given the last morphine treatment. The brains were then removed and post-fixed in 4% paraformaldehyde overnight. All brains were cut in 40–50 μm coronal sections on a vibration microtome (Leica). The rats with a wrong injection site were not included in data analysis.

### Triple-immunofluorescence and quantitative analysis

Sections containing the BLA were incubated for 2 h at 4 °C in a blocking solution containing 10% normal goat serum and 0.2% Triton X-100 in PBS, and then incubated overnight at 4 °C with primary antibody mouse anti-D1 receptor (1:200) and rabbit anti-VGLUT2 (1:3000), dissolved in the blocking solution. After 2 h incubation at 4 °C with the second antibodies (1:200) Alexa Fluor 488-conjugated goat anti-mouse, Alexa Fluor 647-conjugated goat anti-rabbit, and Cy3-conjugated Streptavidin, sections were mounted on glass slides using aqua-mount mounting medium.

Triple-labeled images from each section of the BLA were obtained using confocal microscopy (Olympus FV1000) with a 60× oil-immersion lens and collected at a resolution of 1024 × 1024 pixels. The level of D1 receptor (D1R) immunoreactivity merged with BDA and VGLUT2 in presynaptic terminals in the BLA was estimated in the form of optical density with the help of the MacBiophotonics Image J software (SCR_003070). The confocal microscope setting was kept the same for all scans when the D1R fluorescence intensity was compared. Series of images were captured from the confocal microscope and converted to 8-bit gray-scale images, then the area and mean grey values of white color clusters were measured by a multi measure of the region of interest using MacBiophotonics Image J software. The optical density (OD) was computed by grey values using the formula: OD = log10 (255/(255 – grey value)). Generally, coronal sections from 6 to 9 rats were used for quantitative analysis and 6 to 8 images of each slice were averaged to determine a value for the white color fluorescence intensity.

### Self-administration

Rats (250–280 g) were anaesthetized with chloral hydrate (400 mg/kg, i.p.) and a cannula was inserted into the jugular vein. The cannula was guided subcutaneously up to the skull, where it was fixed to a metal tube secured onto the skull with small screws and fixed with dental acrylic cement. Details of the procedure have been reported previously [45]. Briefly, testing was performed in a standard conditioning cage (21 cm × 21 cm × 28 cm) placed in a sound-attenuated room. The test cage was equipped with active and passive levers, 2 cm above the floor, and a red light located 4 cm above the active lever. The i.v. cannula of the animals tested was connected to the infusion pump. The pressing of the active lever, marked by the red light, resulted in an i.v. infusion of 0.2 mL fluid (5 mg/mL morphine in saline solution or saline only) during 10 s. A depression of the active lever during this time (10 s) did not change the drug infusion. The pressing of the passive lever had no programmed consequences. The drug-naive animals were placed in the test cage and were allowed to i.v. self-administer a drug solution for 2 h a day (for 7 days). The number of the active and passive lever pressings is recorded by LabState (Anilab Software & Instruments Co., Ltd., China). Rats were perfused with saline and followed by 4% paraformaldehyde in PB 4 h after having been given the last test.

### microRNA microarray

microRNA expression profiling of five chronic morphine-treated rats and five saline-treated rats was performed using the miRCURY Hy3/Hy5 power labeling kit and hybridized on the miRCURY™ LNA Array (v.18.0). Following the washing steps, the slides were scanned using the Axon GenePix 4000B microarray scanner. Scanned images were then imported into GenePix Pro 6.0 software (Axon) for the grid alignment and data extraction. Replicated miRNAs were averaged and the microRNAs that were intensified by ≥ 30 in all samples were chosen to calculate the normalization factor. After the normalization, significant differentially expressed miRNAs were identified through Volcano Plot filtering. Hierarchical clustering was performed to show distinguishable microRNA expression profiling among samples.

### Quantitative RT-PCR for the assay of mRNA and microRNA expression

Total RNA, including mRNA and microRNA, was extracted from tissue using miRNeasy Mini Kit from QIAGEN according to the manufacturer’s manual. Quantitative real-time PCR of the D1 receptor mRNA was performed using SYBR® Premix Ex Taq TM II (TaKaRa) through 40 PCR cycles (95 °C for 10 s, 60 °C for 25 s, and 72 °C for 20 s). The real-time RT-PCR for the quantification of miR-105 was carried out with the microRNA first-strand cDNA synthesis kit (TIANGEN) along with the miR-105 specific primers (TIANGEN). After the reverse transcription, quantitative PCR was performed on the mastercycler ep realplex (Eppendorf) using the miRcute microRNA qPCR detection kit (TIANGEN) through 40 PCR cycles (94 °C for 2 min, 94 °C for 20 s, and 60 °C for 34 s). Single PCR products were verified by assessing that the melting temperature of the product had a single value. The following primers were used: Drd1a, Forward, 5′-GGAGATTACTGCCCTGGCTCCTA-3′; Reverse, 5′-GACTCATCGTACTCCTGCTTGCTG-3′; Act, forward, 5′-GGAGATTACTGCCCTGGCTCCTA-3′ and reverse, 5′-GACTCATCGTACTCCTGCTTGCTG-3′; miRNA105, 5′-GGGGTTCAAGTAATCCAGG-3′; U6, 5′-CTCGCTTCGGCAGCACA-3′. To obtain the fold-change in mRNA and microRNA, data were analyzed using the 2^–ΔCT^ method. The final gene expression levels were normalized to the internal control β-actin mRNA and U6 small nuclear RNA, respectively. The expression data were analyzed with the comparative Ct method [46].

### Plasmids construction

The prediction of Drd1 mRNA as a target of miR-miR-105 was made with TargetScan (SCR_010845), miRanda (SCR_010838), and Pictar (SCR_003343) programs. The wt 3′UTR plasmid of Drd1 was cloned and inserted into the 3′UTR of the Renilla luciferase gene of the psiCHECK2 vector. The mut 3′UTR plasmid of Drd1 was produced by means of site-directed mutagenesis. Drd1 3′UTR wt/mut vectors and miR-105 mimics/inhibitors were obtained from GeneCopoeia Inc.

### Dual luciferase reporter assay

HEK-293 T cells were seeded in 24-well plates 24 h before transfection. For the luciferase assay, 100 nm of NC or miR-105 mimic/inhibitor and 0.3 μg psiCHECK2 vector containing Drd1wt/mut 3′UTR were co-transfected using reagent EndoFectinTM Lenti (GeneCopoeia, Inc.). After 48 h, cells were harvested and lysed, and luciferase assays were performed on the Modulus microplate (Turner Biosystem) using the dual-luciferase reporter assay system (Promega) according to the manufacturer’s instructions. These experiments were repeated at least three times in triplicates. The relative luciferase activities were determined by calculating the ratio of Renilla luciferase activities over firefly luciferase activities.

### Western blotting

Immunoblot analysis of D1 receptor was performed on the cultured mPFC cells (extracted from rats born within 24 h) transfected with miR-105 mimic, miR-105 mimic negative control (miR-105 NC), miR-105 inhibitor, and miR-105 inhibitor negative control (miR-105 inhibitor NC) on the eighth day of culture. The cell pellets were homogenized in a buffer containing 100 mM Tris-HCl (pH 6.7), 1% SDS, 143 mM 2-mercaptoethanol, and 1% protease inhibitor. The lysate was centrifuged at 12,000 rpm for 10 min at 4 °C. Protein concentrations were determined using a BCA kit (Pierce Chemicals). The samples were treated with the SDS sample buffer at 95 °C for 5 min, loaded on a 10% SDS polyacrylamide gel and blotted to a PVDF membrane. Each blot was incubated with a rabbit anti-D1 receptor antibody (1:300) or a monoclonal anti-β-actin antibody (1:4000). The horseradish peroxidase development system previously described [47] was used for immunodetection. The immunoreactivity of D1 receptors was normalized to that of β-actin. Each experiment was repeated at least five times.

### Single-cell RT-PCR for the assay of miR-105

Two weeks after the intra-BLA injection of fluorescent microspheres, rats were treated with chronic morphine as described above. Subsequently, single-cell cytoplasmic contents were harvested from the microsphere-labeled neurons in mPFC slices using patch pipettes. Patch pipettes were pulled from capillaries previously heated at 240 °C overnight and filled with RNase-free ACSF buffer (126 mM NaCl, 2.5 mM KCl, 1.25 mM NaH_2_PO_4_, 2 mM MgSO_4_, 2.5 mM CaCl_2_, 25 mM NaHCO_3_, and 10 mM glucose). After establishing the whole-cell patch configuration, the cytoplasm was harvested with a patch pipette by applying a slight negative pressure. The contents were then expelled into a 0.2-mL PCR tube containing ribonuclease inhibitor (10 U) in normal ACSF and quickly cooled on ice. The harvested cytoplasmic pool was processed for reverse transcription using miScript II RT Kit (Cat No. 218160, Qiagen), followed by pre-amplification with 12 cycles (denaturation 94 °C for 30 s, annealing/extension 60 °C for 3 min) for selected targets using miScript PreAMP PCR kit (Cat No. 331451, Qiagen), according to the manufacturer’s protocol. Subsequently, real-time PCR for the quantification of miR-105 was performed on the Mastercycler ep realplex (Eppendorf) using miScript SYBR Green PCR kit (Cat No. 218073, Qiagen, miR-105- or U6-specific primer supplied by Qiagen) through 40 real-time cycles according to the program (denaturation, 94 °C for 15 s; annealing, 55 °C for 30 s; extension, 70 °C for 30 s). Single-cell RT-PCR products were verified by assessing that the melting temperature of the product had a single value.

### MiR-105 lentivirus vector and lentivirus transduction

The miR-105 inhibitor or negative control lentiviral vector PlKD-CMV-GFP-U6-miRNAi containing a CMV-driven eGFP expression cassette was constructed by Genepharma Company. The single-stranded DNA oligonucleotides were designed as follows: (1) pre-miR-105 (“top strand” oligo: gatccGACGGCGCTAGGATCATCAACACCACAGACATCATCTTG AGCACTTGCAAGTATTCTGGTCACAGAATACAACACCACAGACATCATCTTGAGCACTTGCAAGATGATCCTAGCGCCGTCTTTTTTg) and its complementary chain (“bottom strand” oligo: aattcAAAAAAGACGGCGCTAGGATCATCTTGCAAGTGCTC AAGATGATGTCTGTGGTGTTGTATTCTGTGACCAGAATACTTGCAAGTGCTCAAGATGATGTCTGTGGTGTTGATGATCCTAGCGCCGTCg); (2) negative control (“top strand” oligo: tgctgAAATGTACTGCGCGTGGAGACGTTTTGGCCACTGACTGACGT CTCCACGCAGTACATTT) and its complementary sequence (“bottom strand” oligo: cctgAAATGTACTGCGTGGAGACGTCAGTCAGTGGCCAAAACGTCTCCACGCGCAGTACATTTc). The miR-105 overexpression lentivector Ubi-EGFP-MCS containing an Ubi-driven eGFP expression cassette and a miR-105 precursor were constructed by Genechem. The miR-105 precursor sequence was: 5′-CTGTGTACTTTGCTAATAATATGAGTTTCTCTCTCACTTGTCATTGCTGTCTACTACTGTAACATGGCATTAACACCTGTTGTTCTCTCTGTGTGTATTGTAGTCAAGTGCTCAGATGTCTGTGGTGGCTGCTTATGTATCACGGATGTTTGAGCATGTGCTATGGTATCTACTTTTACAACATTGCCATCTGCTTCTGGAACAAAGCCATTCATTACTCTGTTTTAAAACCATGTTCCATGTCTGTCTGTCAGGAGAATCACAGCATATTGT-3′. The negative control lentiviral vector was an eGFP vector without any miRNA sequence. miR-105 inhibitor lentivirus (miR-105 inhibitor-LV, 4 × 10^8^ TU/mL), miR-105 inhibitor negative control lentivirus (miR-105 inhibitor NC-LV, 3 × 10^8^ TU/mL), miR-105 overexpression lentivirus (miR-105-LV, 4 × 10^8^ TU/mL), and miR-105 negative control lentivirus (miR-105 NC-LV, 4 × 10^8^ TU/mL) were stereotaxically injected into mPFC (0.5 μL each side). Three to four weeks later, rats were treated with morphine as described above. Then, brains were fixed and cut in coronal sections. The sections containing BLA were incubated for 2 h at 4 °C in a blocking solution containing 10% normal goat serum and 0.2% Triton X-100 in PBS and incubated overnight at 4 °C with primary antibody mouse anti-D1 receptor (1:200), rabbit anti-eGFP (1:300), and guinea pig anti-VGLUT2 (1:3000) dissolved in the blocking solution. After 2 h incubation at 4 °C with the second antibodies (1:200) Cy3-conjugated goat anti-mouse, Alexa Fluor 488-conjugated goat anti-rabbit, and Alexa Fluor 647-conjugated goat anti-guinea pig, the sections were mounted on glass slides using aqua-mount mounting medium. The acquisition of triple-labeled images and the quantification of the dopamine D1 receptor intensity were the same as above.

### In vitro optogenetic approach for electrophysiology

Bilateral injections of purified and concentrated AAV-CaMKIIα-ChR2-mCherry virus (2.45 × 10^12^ vector genomes/mL, Neuron Biotech Company, China) were stereotaxically performed in 3-week-old male C57BL/6 J mice. Each side of mPFC (AP, +1.95 mm; ML, ±0.35 mm; DV, −2.4 mm) was injected with 0.5 μL AAV for 10 min followed by an additional 10 min to allow the diffusion of viral particles away from the injection site. The virus was allowed to express for a minimum of 6 weeks in order to allow time for sufficient opsin accumulation in the axons. Mice were then treated with morphine as described above. In chronic morphine-treated mice, brain slices were prepared 4 h after the last injection of morphine and maintained in vitro in 5 μM morphine throughout the experiments. Mice were anesthetized and coronal sections of 250 μm containing the mPFC or the BLA were cut on a vibratome (Leica, Germany) and transferred to a normal ACSF at 32 °C (containing 126 mM NaCl, 2.5 mM KCl, 1.25 mM NaH_2_PO_4_, 2 mM MgSO_4_, 2.5 mM CaCl_2_, 25 mM NaHCO_3_, and 10 mM glucose). The viral injection sites in mPFC slices were checked and the animals on which the injection site was outside of the mPFC were removed.

BLA slices were prepared for the optical stimulation and whole-cell voltage-clamp recording. Pyramidal cells of the BLA were visualized using infrared differential interference contrast and fluorescent microscopy. Whole-cell voltage-clamp recordings were made using patch electrodes (4–5 MΩ) containing 130 mM K-gluconate, 8 mM NaCl, 0.1 mM CaCl_2_, 0.6 mM EGTA, 2 mM ATP.Mg, 0.1 mM GTP.Na_3_, and 10 mM HEPES (pH 7.4). The data were collected by EPC10 amplifier and PatchMaster 2.54 software (HEKA, Germany, SCR_000034). For the optical stimulation of mPFC-to-BLA-specific glutamatergic terminals, the ChR2 expressed in the glutamatergic terminals was stimulated by flashing a 470-nm light (5 ms pulses, 0.1 Hz), which was delivered via an optical fiber (core diameter 200 μm, NA = 0.39, ThorLabs, USA) coupled to an LED light source (Mightex, USA) 500 μm above the recording cell. The optical stimulating pulses were given at 0.1 Hz with the light intensity adjusted to evoke an EPSC of approximately 100–200 pA. The light-evoked EPSCs were recorded for 10 min followed by the bath application of 10 μM SKF38393 for an additional 20 min. To observe PPF, two synaptic responses were evoked by a pair of optical stimulating pulses given at short intervals (50 ms) at 0.1 Hz. To observe AMPA and NMDA receptor currents, recording electrodes (4–5 MΩ) were filled with 120 mM caesium methansulphonate, 10 mM HEPES, 0.4 mM EGTA, 2.8 mM NaCl, 5 mM tetraethylammonium chloride, 2.5 mM ATP.Mg, and 0.25 mM GTP.Na_3_ (pH 7.4). The AMPAR/NMDAR ratio was calculated by averaging 20 EPSCs at +40 mV before and after application of the NMDAR blocker AP5 (50 μM) for 5 min. NMDAR responses were calculated by subtracting the average response in the presence of AP5 from that seen in its absence as similar to previous studies [24]. The light intensity of the LED was not changed during the experiments. Series resistance compensation was not used, but cells with an Rs change of more than 20% were discarded.

### Conditioned place aversion (CPA)

The procedure for the CPA test was similar to that described previously [8, 26]. CPA was conducted with a three-compartment place conditioning apparatus (Med Associates, USA). On day 0, the rats were given a preconditioning test (Pretest). The animals were placed in the middle neutral area and were allowed to freely access both sides of the apparatus for 15 min. Rats with a strong preference (>80%) for any compartment were discarded. Before conditioning, morphine dependence was induced by twice daily intraperitoneal injections of morphine at 08.00 and 18.00, as described above. On day 5, 3 h after 40 mg/kg morphine administration, rats were confined to either compartment for 20 min immediately after the subcutaneous injection of naloxone (0.1 mg/kg). On day 6, rats were confined to the opposite compartment for 20 min after the subcutaneous injection of saline. The entire procedure of conditioning was repeated on the following 2 days. The post-test was conducted 24 h after conditioning on day 9. The rats were allowed to freely explore the three compartments for 15 min and CPA score was calculated as the difference between the time spent in the saline-paired compartment and the time spent in the naloxone-paired compartment (the time in the naloxone-paired compartment minus the time in the saline-paired compartment).

### Double-immunofluorescence and quantitative analysis of c-fos in projection neurons from the mPFC to the BLA

After the rats were anesthetized with chloral hydrate (400 mg/kg, i.p.) and placed in a stereotaxic device (Stoelting), retrograde tracer fluorogold (FG, 4% dissolved in saline) was injected into the BLA (AP, −2.6 mm; ML, ±5.0 mm; DV, −8.5 mm, based on the atlas of Paxinos and Watson, 2005) [44] in a 0.5-μL volume on each side of the brain and retained in place for an additional 10 min to optimize diffusion. Rats were given 7 days to recover and morphine dependence was induced by intraperitoneal injections of morphine as described above before undergoing CPA procedures. A half hour after the post-conditioning test, rats were anesthetized with chloral hydrate (400 mg/kg, i.p.) and perfused with 4% paraformaldehyde in PB. The brains were removed and fixed overnight and were then cut into 40-μm coronal sections using a vibration microtome (Leica). To verify the location of the injection site, coronal sections containing the BLA were examined under a fluorescence microscope. Rats with an incorrect injection site were excluded from the study. Sections containing mPFC were incubated with rabbit anti-c-fos antibody (1:400) overnight and incubated with biotinylated goat anti-rabbit antibody (1:200) for 2 h at 4 °C followed by Cy3-conjugated Streptavidin (1:200) for 40 min at room temperature. Biotin was blocked using a Streptavidin/Biotin Blocking Kit (Vector Laboratories), according to the manufacturer’s manual. The sections were incubated with rabbit anti-FG antibody (1:3000) overnight at 4 °C and then incubated with Alexa Fluor 488-conjugated goat anti-rabbit antibody (1:200) for 2 h at 4 °C. The sections were mounted on glass slides using aqua-mount mounting medium. All antibodies were dissolved in PBS with 10% normal goat serum and 0.2% Triton X-100. A series of slices containing the mPFC were imaged by fluorescence microscopy with a 4× magnification lens. Quantification of c-Fos-, FG-, or c-Fos + FG-labeled neurons was performed with MacBiophotonics Image J software with the same threshold. The positive cells were defined with staining above the basal background. Counts collected from six sections from each rat were averaged to produce a value.

### Drugs

Triton X-100, PBS, rabbit anti-VGLUT2 antibody (V2514), SKF38393, DMSO, ATP.Mg, GTP.Na_3_, HEPES, EGTA, AP5 and DNQX were purchased from Sigma. BDA, Trizol reagent, glutamax, B27, lipofectamine RNAiMAX Transfection Reagent, and ribonuclease inhibitor were from Invitrogen. Morphine was obtained from Shenyang No. 1 Pharmaceutical Factory. Aqua-mount mounting medium was purchased from Thermo Fisher Scientific. Goat serum, Alexa Fluor 488-conjugated goat anti-mouse antibody (Code: 115-545-166), Alexa Fluor 647-conjugated goat anti-rabbit antibody (Code: 111-605-144), Cy3-conjugated streptavidin antibody (Code: 016-160-084), Cy3-conjugated goat anti-mouse antibody (Code: 115-165-003), Alexa Fluor 488-conjugated goat anti-rabbit antibody (Code: 115-545-003), Alexa Fluor 647-conjugated goat anti-guinea pig antibody (Code: 106-605-003), and biotinylated goat anti-rabbit antibody (Code: 111-065-008) were purchased from Jackson ImmunoResearch Laboratories. Neurobasal-A medium, 10% fetal calf serum, and 0.4% Pen/Strep solution were obtained from GIBCO. Risbase and EDTA were purchased from Tocris. Rabbit anti-eGFP antibody (04-337) and guinea pig anti-VGLUT2 antibody (AB2251-I) were from Millipore. Rabbit anti-FG antibody (ab36050) and rabbit anti-D1 receptor antibody (ab40653) were obtained from Abcam. Monoclonal anti-β-actin-HRP antibody (#5125) and rabbit anti-c-Fos antibody (#5348) were purchased from Cell Signaling Technology and mouse anti-D1 receptor antibody (sc14001) from Santa Cruz Technology. FG (52-9400) was purchased from Fluorochrom. Other reagents in AR grades were products of Shanghai Chemical Plant. DNQX was dissolved in DMSO and the remaining reagents were dissolved in ddH_2_O. When DMSO was used as the vehicle, drugs were initially dissolved in 100% DMSO and then diluted into ACSF at a final DMSO concentration of less than 0.5%. SKF38393 was dissolved in distilled water just before use.

### Off-line data analysis

Off-line data analysis was performed using a PatchMaster (HEKA, SCR_000034) and SigmaPlot (Jandel Scientific, SCR_003210). The averaged 30 raw traces taken during the last 5 min of baseline and last 5 min of drug application were shown as the typical traces of light-evoked EPSC. PPF = EPSC2/EPSC1. Numerical data were expressed as mean ± SE. Statistical significance was determined using Student’s *t* test for comparisons between two groups or ANOVA followed by Student–Newman–Keuls test for comparisons among three or more groups. In all cases, n refers to the number of cells or animals. In the patch clamp studies, every cell was from a different slice and a group of cells in each experiment was from at least three animals.

## Additional files

Additional file 1: Influence of Chronic morphine on microRNA expression. (XLS 1725 kb)

Additional file 2: Data values for all experiments where n<6. (DOCX 17 kb)

## Abbreviations

AMPA: α-amino-3-hydroxy-5-methyl-4-isoxazolepropionic acid
BDA: biotinylated dextran amine
BLA: basolateral amygdala
ChR2: channelrhodopsin-2
CPA: conditioned place aversion
EPSC: excitatory postsynaptic currents
FG: fluorogold
LV: lentiviral vector
mPFC: medial prefrontal cortex
PPF: paired-pulse facilitation
VGLUT2: vesicular glutamate transporters 2
