# Supplementary material for: Memory retrieval in addiction: a role for miR-105-mediated regulation of D1 receptors in mPFC neurons projecting to the basolateral amygdala
Source: BMC Biol. 2017 Dec 27;15:128. doi: 10.1186/s12915-017-0467-2 (PMC5745965; doi:10.1186/s12915-017-0467-2)
Supplement: Supplementary file 2 — Data values for all experiments where n<6. (DOCX 17 kb) [file 12915_2017_467_MOESM2_ESM.docx]

**Raw data for all experiments where n<6**

Raw data in 3 of Fig. 4B (n = 4)

| Group | Saline | Morphine |
| --- | --- | --- |
| 1 | 128.6 | 79.6 |
| 2 | 67.2 | 112.4 |
| 3 | 135.2 | 84.2 |
| 4 | 69.0 | 88.8 |
| Mean | 100.0 | 91.3 |
| SEM | 18.5 | 7.3 |
| Paired *t* test | *P* = 0.743 | |

Raw data in 4 of Fig. 4B (n = 5)

| Group | Saline | Morphine |
| --- | --- | --- |
| 1 | 48.1 | 91.7 |
| 2 | 181.9 | 200.9 |
| 3 | 43.3 | 93.1 |
| 4 | 177.3 | 173.9 |
| 5 | 49.4 | 53.5 |
| Mean | 100.0 | 122.6 |
| SEM | 32.5 | 27.7 |
| Paired *t* test | *P* = 0.098 | |

Raw data in bottom panel of Fig. 4C

|  | wt 3’UTR of D1 | | mut 3’UTR of D1 | |
| --- | --- | --- | --- | --- |
| Group | miRNA NC | miR-105  mimics | miRNA NC | miR-105  mimics |
| 1 | 0.88 | 0.69 | 2.30 | 2.12 |
| 2 | 0.91 | 0.67 | 2.18 | 2.08 |
| 3 | 0.93 | 0.67 | 2.02 | 2.19 |
| Mean | 0.91 | 0.68 | 2.17 | 2.13 |
| SEM | 0.01 | 0.01 | 0.08 | 0.03 |
| Paired *t* test | *P* = 0.009 | | *P* = 0.75 | |

Raw data in Fig. 5A (n = 5)

| Group | Saline | Morphine |
| --- | --- | --- |
| 1 | 88.4 | 194.0 |
| 2 | 27.0 | 68.0 |
| 3 | 179.3 | 275.0 |
| 4 | 103.1 | 208.7 |
| 5 | 102.2 | 190.8 |
| Mean | 100.0 | 187.3 |
| SEM | 24.3 | 33.5 |
| Paired *t* test | *P* = 0.0019 | |

Raw data in Fig. 5B (n = 5)

| Group | Saline | Morphine |
| --- | --- | --- |
| 1 | 194.2 | 141.6 |
| 2 | 27.0 | 21.6 |
| 3 | 57.8 | 39.7 |
| 4 | 57.8 | 24.2 |
| 5 | 163.2 | 141.6 |
| Mean | 100.0 | 73.7 |
| SEM | 33.0 | 27.9 |
| Paired *t* test | *P* = 0.03 | |

Raw data in right panel of Fig. 6A (n = 5)

| Group | Saline | Morphine |
| --- | --- | --- |
| 1 | 184.1 | 58.1 |
| 2 | 62.2 | 16.0 |
| 3 | 46.2 | 45.5 |
| 4 | 93.0 | 47.3 |
| 5 | 114.5 | 40.9 |
| Mean | 100.0 | 41.6 |
| SEM | 24.1 | 7.0 |
| Paired *t* test | *P* = 0.047 | |

Raw data in right panel of Fig. 6B (n = 5)

| Group | miR-105 inhibitor NC-LV | miR-105 inhibitor-LV |
| --- | --- | --- |
| 1 | 93.8 | 310.1 |
| 2 | 111.8 | 178.9 |
| 3 | 154.5 | 353.4 |
| 4 | 82.2 | 245.8 |
| 5 | 57.7 | 85.7 |
| Mean | 100.0 | 243.8 |
| SEM | 16.2 | 47.5 |
| Independent *t* test | *P* = 0.028 | |

Raw data in middle panel of Fig. 7C (n = 5)

| Group | Control | SKF38393 |
| --- | --- | --- |
| 1 | 1.13583 | 1.52673 |
| 2 | 1.2111 | 1.07787 |
| 3 | 1.33917 | 1.11488 |
| 4 | 1.16732 | 1.10581 |
| 5 | 0.97269 | 0.93291 |
| Mean | 1.16522 | 1.15164 |
| SEM | 0.05929 | 0.09936 |
| Paired *t* test | *P* = 0.904 | |

Raw data in right panel of Fig. 7C (n = 5)

| Group | Control | SKF38393 |
| --- | --- | --- |
| 1 | 1.23236 | 0.94973 |
| 2 | 1.57707 | 1.17459 |
| 3 | 1.18084 | 0.8816 |
| 4 | 1.07693 | 0.91406 |
| 5 | 1.28434 | 1.28728 |
| Mean | 1.27031 | 1.04145 |
| SEM | 0.08401 | 0.08011 |
| Paired *t* test | *P* = 0.0299 | |

Raw data in Fig. 10B (n = 5)

| Group | miR-105 NC-LV | miR-105-LV |
| --- | --- | --- |
| 1 | 64.8 | 51.1 |
| 2 | 87.4 | 35.1 |
| 3 | 127.0 | 56.7 |
| 4 | 108.1 | 71.6 |
| 5 | 112.7 | 83.0 |
| Mean | 100.0 | 59.5 |
| SEM | 10.8 | 8.3 |
| Independent *t* test | *P* = 0.018 | |
